# Transcriptome analysis reveals the difference between “healthy” and “common” aging and their connection with age‐related diseases

**DOI:** 10.1111/acel.13121

**Published:** 2020-02-19

**Authors:** Lu Zeng, Jialiang Yang, Shouneng Peng, Jun Zhu, Bin Zhang, Yousin Suh, Zhidong Tu

**Affiliations:** ^1^ Department of Genetics and Genomic Sciences Icahn School of Medicine at Mount Sinai New York New York; ^2^ Department of Genetics Albert Einstein College of Medicine New York New York

**Keywords:** age‐related diseases, common aging, gene expression, GTEx, healthy aging, human aging signatures, unhealthy aging

## Abstract

A key goal of aging research was to understand mechanisms underlying healthy aging and develop methods to promote the human healthspan. One approach is to identify gene regulations unique to healthy aging compared with aging in the general population (i.e., “common” aging). Here, we leveraged Genotype‐Tissue Expression (GTEx) project data to investigate “healthy” and “common” aging gene expression regulations at a tissue level in humans and their interconnection with diseases. Using GTEx donors' disease annotations, we defined a “healthy” aging cohort for each tissue. We then compared the age‐associated genes derived from this cohort with age‐associated genes from the “common” aging cohort which included all GTEx donors; we also compared the “healthy” and “common” aging gene expressions with various disease‐associated gene expressions to elucidate the relationships among “healthy,” “common” aging and disease. Our analyses showed that 1. GTEx “healthy” and “common” aging shared a large number of gene regulations; 2. Despite the substantial commonality, “healthy” and “common” aging genes also showed distinct function enrichment, and “common” aging genes had a higher enrichment for disease genes; 3. Disease‐associated gene regulations were overall different from aging gene regulations. However, for genes regulated by both, their regulation directions were largely consistent, implying some aging processes could increase the susceptibility to disease development; and 4. Possible protective mechanisms were associated with some “healthy” aging gene regulations. In summary, our work highlights several unique features of GTEx “healthy” aging program. This new knowledge could potentially be used to develop interventions to promote the human healthspan.

## Graphical abstract

A key goal of aging research was to study mechanisms underlying healthy aging and based on them to identify effective methods to promote human healthspan. Here, we leveraged transcriptome data from GTEx to investigate gene expression profiles of both “healthy” and “common” aging in humans and its connection with diseases. Our results highlighted unique features of “healthy” aging and elucidated an intimate intertwined relationship among “healthy” aging, “common” aging, and age‐related diseases.

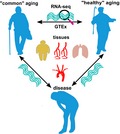

## 1 INTRODUCTION

Aging is the major risk factor for many age‐related diseases, including cancers, metabolic diseases, neurodegenerative diseases, and cardiovascular diseases (Johnson, Dong, Vijg, & Suh, 2015). The prevalence of age‐related comorbidity is high, as over 80% of the elderly population have at least one chronic disease (CDC, 2011). Helping this 80% of the aging population to live with improved health has become a major task for human aging and geroscience research, which can have major impact to socioeconomics and humanity.

It remains elusive why the fortunate 20% of individuals above 65 years of age can live without any major health issues while the rest of us have to endure one or more chronic course of illness. Since very long‐lived individuals (e.g., centenarians) tend to have a lower incidence of chronic illness than those in their 80s and 90s (Kheirbek et al., 2017), and longevity is heritable with an estimated heritability around 25%, this suggests that healthy aging is not a random event, but that there are underlying biological mechanisms favorably interplayed with certain environmental factors.

Gene expression and other types of “omics” data have been widely used to study the process of aging (Edwards et al., 2007; Peters et al., 2015). For examples, epigenome and transcriptome landscapes with aging in mice have revealed widespread induction of inflammatory responses (Benayoun et al., 2019); likewise, the downregulation of mitochondrial genes across human tissues has been consistently reported (Glass et al., 2013; Yang et al., 2015). Studying transcriptomes across multiple species with varied lifespans has similarly reveled a potential role for gene expression regulation in contributing to longer lifespans (S. Ma et al., 2018). In addition, epigenetic clock of aging has been developed based on DNA‐methylation markers (Horvath & Raj, 2018). These studies support that gene expression and epigenetic regulations can inform our understanding of human aging.

Despite recent development in transcriptomic and epigenetic aging research, limited work has been performed to study human healthy aging. To investigate how healthy aging is different from aging in the general population at a systems level, one approach is to profile tissues from a healthy aging cohort and compare with tissue profiles from common aging population, to identify gene regulations unique to the healthy aging. However, obtaining essential tissues from healthy individuals is very challenging for ethical and practical reasons. On the other hand, several large‐scale human genomic datasets are available and could be repurposed for aging research. Here, we leveraged GTEx data (Consortium, 2017) to investigate the potential difference between “healthy” and “common” aging in humans and study their connection with diseases.

## 2 RESULTS

### 2.1 Identifying age‐associated genes using GTEx data

We obtained gene expression and genotype data from GTEx v7/v8 and examined 46 tissues with more than 80 samples per tissue type (Tables S1 and S2, Figures S1 and S2). Age‐associated gene expressions were identified using a previously established regression model (Yang et al., 2015). Post‐mortem interval (PMI) was adjusted as an additional covariate (Equation 1), based on the evidence that some gene expressions continued to change after donor death (Ferreira et al., 2018). Further analysis suggested that identified aging genes based on PMI adjustment were more comparable with previous independent studies (Table S3). Adjusting donor's death time in the day only slightly changed the result compared with PMI adjustment (Figure S3) and was not included in the final model. For the 46 tissue types, tibial artery showed the largest number of age‐associated genes (*n* = 8,709) (we called them as aging genes for brevity, using FDR<=0.01 as a cutoff), followed by aorta artery (*n* = 5,826), skeletal muscle (*n* = 4,444), nerve tibial (*n* = 3,619) and subcutaneous fat (*n* = 3,812). In contrast, very few aging genes were identified in brain‐spinal cord (*n* = 0), pituitary (*n* = 1), small intestine‐terminal ileum (*n* = 1) and liver (*n* = 7) (Table S4).

### 2.2 GTEx human aging signatures recapitulated aging genes identified from other independent studies

To evaluate whether aging signatures derived from GTEx were reproducible, we compared our results with aging gene lists from five independent studies covering multiple tissue types: brain (Berchtold et al., 2008), skin (Glass et al., 2013), adipose (Glass et al., 2013), blood (Lu et al., 2018), and lung (de Vries et al., 2017). We found that aging genes identified from GTEx showed a significant overlap with aging genes from these independent studies. For example, in brain and lung tissues, more than 30% of GTEx aging genes were also found in other independent brain and lung aging studies, with *p*‐values of 5.81 × 10^−176^ and 4.32 × 10^−59^, respectively (Table S3).

To further evaluate the overlap of GTEx aging genes with other studies, we collected additional age‐associated gene expression studies in human brain tissues (Kumar et al., 2013; Rhinn & Abeliovich, 2017; Twine, Janitz, Wilkins, & Janitz, 2013). We found that GTEx brain aging signatures generally shared higher similarity with other brain studies (Table S5) compared with the similarity among these brain studies themselves. Therefore, our results suggested that the aging signatures from GTEx can recapitulate and are largely comparable with the aging signatures from previous independent studies.

### 2.3 Defining GTEx “healthy” aging samples

One approach of studying human healthy aging is to profile tissue samples from a healthy aging cohort and compare with tissue profiles from the general aging population to identify features such as gene expression regulations that are unique to the healthy aging cohort. Although this may appear to be a routine experiment, it is actually very challenging to implement due to the difficulty in obtaining essential tissues (liver, heart etc.) from truly healthy individuals. To collect essential tissues from healthy individuals, it is only feasible when such individuals die from lethal accidents. As lethal accidents happen at a low frequency and are highly unpredictable, ascertaining donors' healthy status at the time of accident and prompt tissue collection from hundreds of donors right after death will be very challenging to achieve. On the other hand, repurposing existing human genomic data for aging research represents a convenient alternative. Since GTEx provides disease annotation for each donor (e.g., ischemic heart disease, chronic respiratory disease, and hypertension), it could be used to study the difference between “common” and “healthy” aging at a transcriptome level. However, such convenience is accompanied with some limitations when used for studying human healthy aging. It is of note that individuals not annotated with any disease may not necessarily be truly healthy. For example, a prediabetic individual may not be annotated for diabetes but should not be considered healthy in a strict‐sense. Despite this limitation, we think it is possible to identify relatively healthy and/or unhealthy individuals/tissues based on donors' disease annotations, and we explored different strategies to define GTEx “healthy” aging cohorts.

The first approach is to filter out donors annotated with one or more diseases, and count the remaining individuals as the “true” healthy cohort (we call it GTEx “disease‐free” cohort). Although this may appear to be reasonable to define GTEx healthy cohort, it has several limitations: 1. As aforementioned, an individual not annotated with any diseases is not necessarily truly healthy; 2. Rather limited number of samples are left for each tissue type when we filter out donors with one or more diseases; 3. The age distribution of this “disease‐free” cohort is quite different from the overall age distribution (Figure S4) as it includes more younger donors. A second approach is to identify relatively healthy samples for each tissue type. Specifically, for a tissue type, we determined disease categories relevant to the tissue, for example, we considered various lung diseases for the lung tissue; diabetes and obesity for the adipose tissue. Tissues from donors that were not annotated with the corresponding diseases are considered “healthy” (called “tissue‐level healthy”). Similarly, we used different methods to stratify a disease cohort. The first method is to consider all the donors excluding “disease‐free” donors (called “disease” cohort), and the second method is to consider donors who were annotated with diseases relevant to the specific tissue (called “tissue‐level disease”). We evaluated both approaches by comparing “healthy” samples with “disease” samples to derive differentially expressed genes, and cross‐check with other independent disease gene expression signatures, which we describe in the following section.

### 2.4 Differentially expressed genes between GTEx “tissue‐level healthy” versus*.* “tissue‐level disease” best reproduce disease signatures from independent disease transcriptome studies

A good definition of healthy/disease samples should allow us to derive disease signatures that are comparable with disease signatures from other independent studies. We calculated disease differentially expressed genes (DEGs) between “disease‐free” versus*.* “disease,” “disease‐free” versus*.* “tissue‐level disease,” “tissue‐level healthy” versus*.* “tissue‐level disease” in subcutaneous fat and compared them with disease signatures from prior independent studies. As shown in Table T2, the “disease‐free” versus*.* “disease” did not identify many DEGs, while the “tissue‐level healthy” versus*.* “tissue‐level disease” reported the largest number of DEGs which showed the strongest overlap enrichment with prior disease signatures (see details of the comparison in Text S1). Given the aforementioned limitation of the “disease‐free” cohort (i.e., not necessarily true healthy, limited sample size, and biased age distribution), we consider GTEx “healthy” aging defined at tissue level is a reasonable choice.

**Figure 1:**
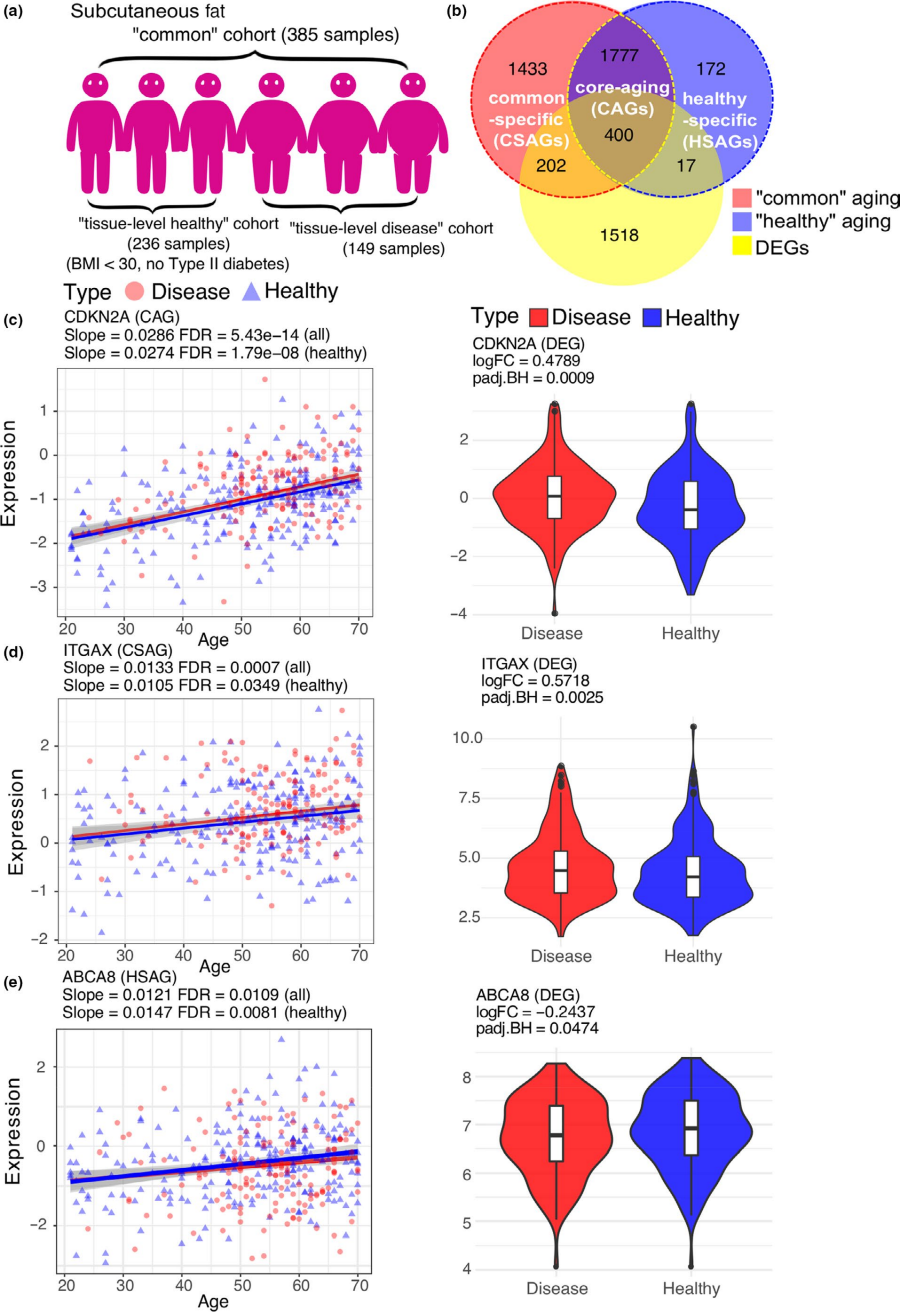
Examples of age‐associated gene expression changes in GTEx subcutaneous fat. (a) Cartoon illustration of separating donors into the “tissue‐level healthy,” “common,” and “tissue‐level disease” cohorts. (b) Venn diagram shows the relationship among “common” aging genes, “healthy” aging genes, and DEGs calculated between the “tissue‐level healthy” cohort and the “tissue‐level disease” cohort in subcutaneous fat. (c, d, e) Scatter plots show three representative age‐associated gene expression patterns in three gene sets (from top to bottom: CAGs, CSAGs, or HSAGs). The red line and dots denote the regression line and gene expression levels for the “disease” cohort, the blue color is for the “healthy” cohort. Violin plots show the gene expression differences between “healthy” (blue) and “disease” (red) individuals for the corresponding genes displayed in the scatter plots

To better define a “tissue‐level healthy” cohort, we required a tissue to have a relatively large number of aging genes, and the sample size of “tissue‐level disease” to be also relatively large (i.e., >20% of samples need to be from disease donors). Four tissue types, namely subcutaneous fat, tibial artery, aorta artery, and lung met these criteria and were selected for further analysis. In subcutaneous fat (total *n* = 385), the “healthy” aging signatures were calculated from donors without type 2 diabetes (T2D) and body mass indexes (BMIs) <30 (*n* = 236) (Figure 1a). In tibial artery (total *n* = 382), the “healthy” cohort (*n* = 292) was defined as GTEx donors without ischemic heart disease/heart attack/acute coronary syndrome. In lung (total *n* = 379), the “healthy” cohort (*n* = 257) was defined as donors without chronic respiratory diseases, asthma or pneumonia (Table S6).

As we pointed out previously, the DEGs by comparing “tissue‐level healthy” versus*.* “tissue‐level disease” samples showed the strongest overlap with disease signatures from other independent studies in the corresponding tissues (Table S7). For example, Soronen et al. (Soronen et al., 2012) reported 148 insulin‐resistance related genes from adipose tissue, 81 of them overlapped with GTEx DEGs in subcutaneous fat, with a *p*‐value of 1.23 × 10^−42^. These DEGs showed no significant overlap enrichment in artery or lung, suggesting the disease‐associated genes were highly tissue‐specific. Similarly, genes associated with coronary heart disease (CHD) identified from peripheral whole blood (Joehanes et al., 2013) were exclusively enriched for GTEx tibial artery DEGs (*p*‐value = .03). Obesity‐related genes obtained from fat (Font‐Clos, Zapperi, & La Porta, 2017) were overrepresented only in DEGs from subcutaneous fat and lung (*p*‐values = 7.90 × 10^−4^ and 2.28 × 10^−3^, respectively), but not in tibial artery. Genes involved in COPD detected from lung (Wang et al., 2008) were found strongly enriched for GTEx lung DEGs (*p*‐value = 5.79 × 10^−4^), while not in other tissue types. Together, these comparisons indicated that our definition of GTEx “healthy” aging at tissue level was biologically meaningful.

In addition to the “healthy” and “common” aging cohorts, we also considered an “unhealthy” aging cohort in subcutaneous fat (*n* = 183) to facilitate the comparison. In general, age‐related diseases develop in old population, while young individuals should be mostly free of these diseases. Instead of using “tissue‐level disease” samples to define the “unhealthy” aging cohort, we created an “unhealthy” cohort by combining healthy donors (without type 2 diabetes and BMI < 30) in their 20–40 years and unhealthy donors (with type 2 diabetes and BMI > 30) in their 40–70 years. We excluded young donors with T2D and BMI>=30 because the “disease” in these individuals were not associated with aging, and including these samples impacted our capability of identifying age‐associated genes. In fact, only 58 age‐associated genes were found in the “tissue‐level disease” cohort, while 4,341 age‐associated genes were identified in this “unhealthy” cohort. The choice of age 40 as the cutoff age is arbitrary, but partially due to the fact that GTEx donors older than 40 years are more likely to have age‐related diseases (Figure S4).

### 2.5 Some “common” aging genes could be driven by diseases while some “healthy” aging genes are likely protective genes

To compare “healthy” and “common” aging signatures, we divided aging genes into three groups: aging genes only seen in the “common” cohort (“common‐specific aging genes,” CSAGs), aging genes only observed in the “healthy” cohort (“healthy‐specific aging genes,” HSAGs), and common aging genes identified from both cohorts (“core‐aging genes,” CAGs). In general, we observed a large overlap between “common” and “healthy” aging signatures in all the four tissues we inspected. For example, in subcutaneous fat, 3,812 “common” aging genes overlapped with 2,366 “healthy” aging genes by 2,177 genes (Figure 1b and Table S6), suggesting that there exists a core aging program regardless of the health status of the aging individuals. Similarly, we found “unhealthy” aging (4,341 genes) also shared large number of its gene regulations with “common” (2,724 genes) and “healthy” aging (1,846 genes), despite that “unhealthy” and “healthy” aging genes were derived from different samples (Figure S5).

We provided examples of gene regulation for CAGs, CSAGs, and HSAGs in Figure 1. As an example of CSAGs, the expression of integrin subunit alpha X (*ITGAX*) significantly correlated with age in subcutaneous fat in the “common” and “unhealthy” cohorts (FDR = 7.12 × 10^−4^ and 1.35e × 10^−5^, respectively), while its association with age was much less significant in the “healthy” cohort (FDR = 0.03) (Figure 1d and Figure S5c). *ITGAX* encodes integrin alpha X chain protein (also named CD11c), previous studies reported that CD11c expression in adipose tissue was significantly increased in both diet‐induced obesity mice and humans (Wu et al., 2010). This is consistent with our results as *ITGAX* was upregulated in GTEx adipose tissue of donors with high BMIs and T2D (Figure 1d and Figure S5c), suggesting its upregulation is associated with disease development in “unhealthy” aging. In contrast, as an example of HSAGs, ATP Binding Cassette Subfamily A Member 8 (*ABCA8*) showed strong upregulation with age (FDR = 8.15 × 10^−3^) in the “healthy” cohort (Figure 1e and Figure S5d), while no association was observed in the “unhealthy” cohort (FDR = 0.23). HDLc levels were found decreased by 29% (*p* = .01) in *ABCA8* deficiency mice on a high‐cholesterol diet compared with wild‐type mice (Trigueros‐Motos et al., 2017). ABC transporters protect cells against unrelated (toxic) substances by pumping them across cell membranes (Tang et al., 2010). Lastly, for CAGs, the upregulated expression of cyclin dependent kinase inhibitor 2A (*CDKN2A*) was significantly associated with age (FDR = 5.43 × 10^−14^, 1.79 × 10^−8^ and 1.31 × 10^−9^, respectively) in all three cohorts (Figure 1c and Figure S5b). *CDKN2A* encodes for INK4 family member p16 (or p16^INK4a^) which is a well‐recognized cell senescence marker (Coppé et al., 2011). The increased expression of *CDKN2A* has been suggested as a biomarker of physiological age (Krishnamurthy et al., 2004).

In addition, we found that CAGs and CSAGs had significant higher overlap with disease‐associated DEGs compared to HSAGs. As shown for subcutaneous fat (Figure 1b), 202 and 400 genes from CSAGs and CAGs were disease DEGs (*p*‐values = 5.40 × 10^−3^ and 1.36 × 10^−32^, respectively; Table S8). In contrast, only 17 disease DEGs were also HSAGs (*p*‐value = .78). Similarly, CAGs from tibial artery were specially enriched for its disease DEGs (*p*‐value = 9.73 × 10^−10^), and lung CSAGs were also overrepresented in lung disease DEGs (*p*‐value = 3.09 × 10^−11^). While HSAGs were found much less significantly enriched for disease DEGs in either tibial artery (*p*‐value = .03) or lung (*p*‐value = 1.00).

### 2.6 The direction of aging gene regulation is largely consistent with the regulation direction of genes associated with age‐related diseases

**Figure 2:**
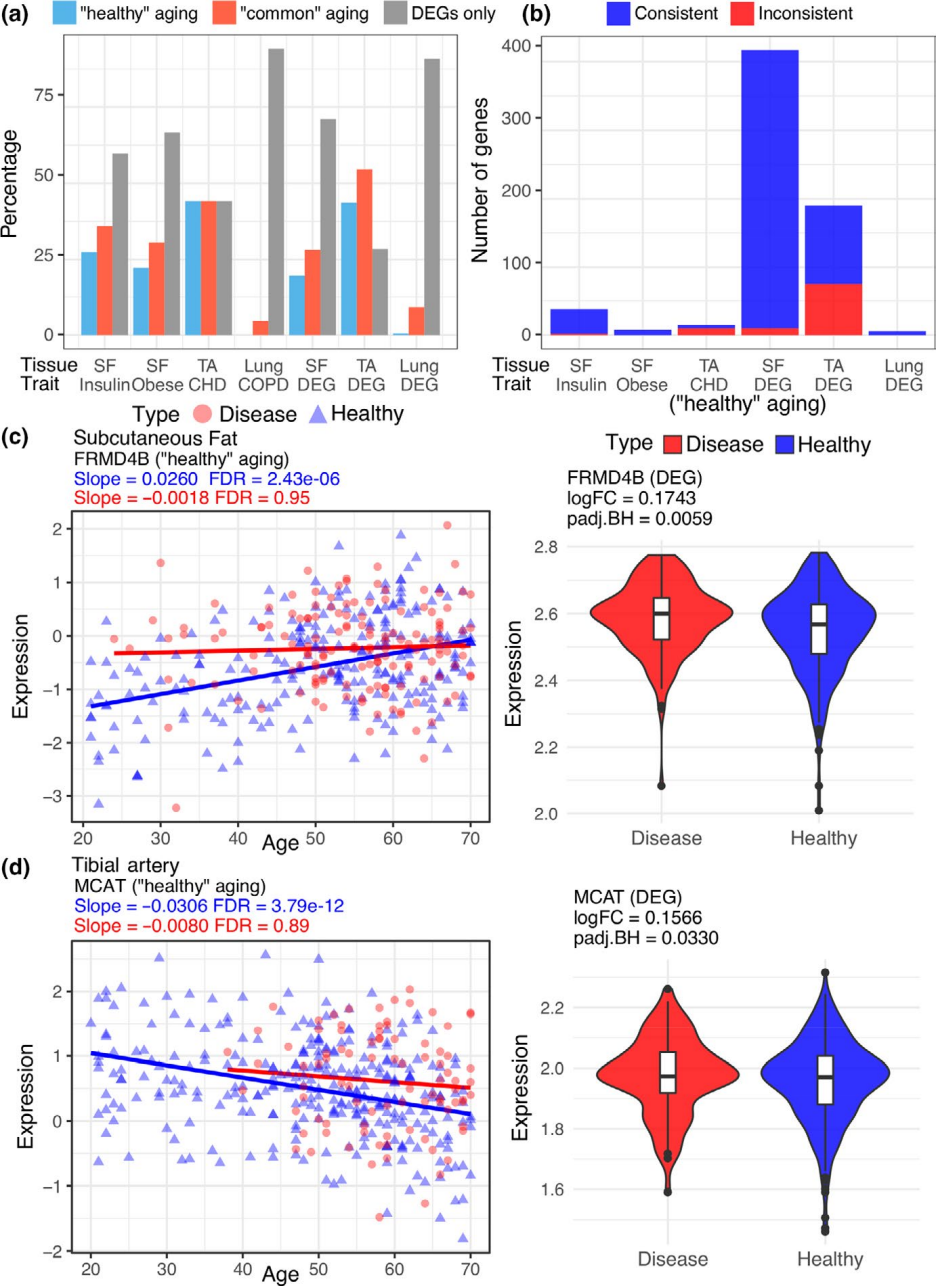
The relationship between age‐associated versus. disease‐associated genes. (a) The percentage of aging genes (blue: “healthy” aging; red: “common” aging) overlapped with disease DEGs and the percentage of disease DEGs that were not associated with age (gray bars). Disease DEGs include four disease signatures from prior work (insulin‐resistance, obesity, coronary heart disease [CHD], and chronic obstructive pulmonary disease [COPD]) and three disease DEGs based on GTEx analysis (simply labeled as DEG). The tissues plotted were subcutaneous fat (SF), tibial artery (TA), and lung. (b) The number of “healthy” aging genes whose direction was consistent (blue) or inconsistent (red) with the direction of gene regulations in 6 disease DEGs as in a (no common genes were found between COPD and “healthy” aging genes in lung). (c) An example of “healthy” aging gene in subcutaneous fat with the same direction of gene expression change as disease DEG. (d) An example of “healthy” aging gene in tibial artery with an opposite direction of gene expression change from disease DEG. The red line and dots denote the regression line and samples for the “disease” cohort, the blue line and dots are for the “healthy” cohort. Violin plots show the gene expression differences between “healthy” (blue) and “disease” (red) individuals

One key question in geroscience is to understand why aging dramatically increases the incidence of various age‐related diseases. We compared our aging signatures with seven disease signatures, four from previous independent studies (insulin‐resistance, obesity, CHD, and COPD) and three from GTEx calculated DEGs. We observed that most of the disease DEGs were not aging genes from GTEx (Table S9). Using insulin‐resistance/obesity‐related DEGs as an example, over 60% of them were not associated with age in subcutaneous fat. Furthermore, as previously noticed, “healthy” aging was less enriched for disease DEGs compared with “common” aging. For example, in subcutaneous fat, 36% of “common” aging genes were found as insulin‐resistance genes, while only 27% of “healthy” aging genes were insulin‐resistance genes (Figure 2a).

Although disease and aging signatures were largely different, a substantial number of gene expression regulations were common between them. We considered it interesting to test if age‐associated gene expression (particularly in the “healthy” aging) would have similar directions as the regulation changes in disease conditions. We decided to focus on the “healthy” aging cohort since the “common” aging cohort contained disease individuals; therefore, the “common” aging gene regulations were not independent from disease‐associated gene regulations. On the other hand, “healthy” aging genes were identified from the “healthy” cohort, which shared no donors with the “disease” cohort. Our results showed that the direction of “healthy” aging gene regulations were largely consistent with the direction of disease DEG regulations (Figure 2b,c). Using FERM domain containing 4B (*FRMD4B*) in subcutaneous fat as an example, it is an inflammation‐related gene whose expression was upregulated in the adipose tissue in insulin‐resistant compared to insulin‐sensitive group (Wiklund et al., 2016). Our results also showed that the gene expression of *FRMD4B* was upregulated with age and disease. This illustrates that even with “healthy” aging, some genes' regulation may promote the tissue to a state that resembles the disease state.

While the direction of gene expression regulation is largely consistent between “healthy” and disease signatures, some gene regulations showed opposite regulation directions (Figure 2d). For example, malonyl‐CoA‐Acyl carrier protein transacylase (*MCAT*), a gene related to pathways like fatty acid metabolism and mitochondrial fatty acid beta‐oxidation, its gene expression was found negatively correlated to plasma HDL levels (Ma, Dempsey, Stamatiou, Marshall, & Liew, 2007). In GTEx data, *MCAT* showed a downregulation in “healthy” aging but was upregulated in disease population in the tibial artery, suggesting the downregulation of this gene could potentially be beneficial for “healthy” aging.

### 2.7 Difference in function enrichment between “healthy” and “common” aging signatures

We investigated the function similarity and difference between “healthy” and “common” aging signatures using DAVID tools (Dennis et al., 2003). Genes were divided into up‐ and downregulation with respect to age and were annotated separately (Table S10).

**Figure 3:**
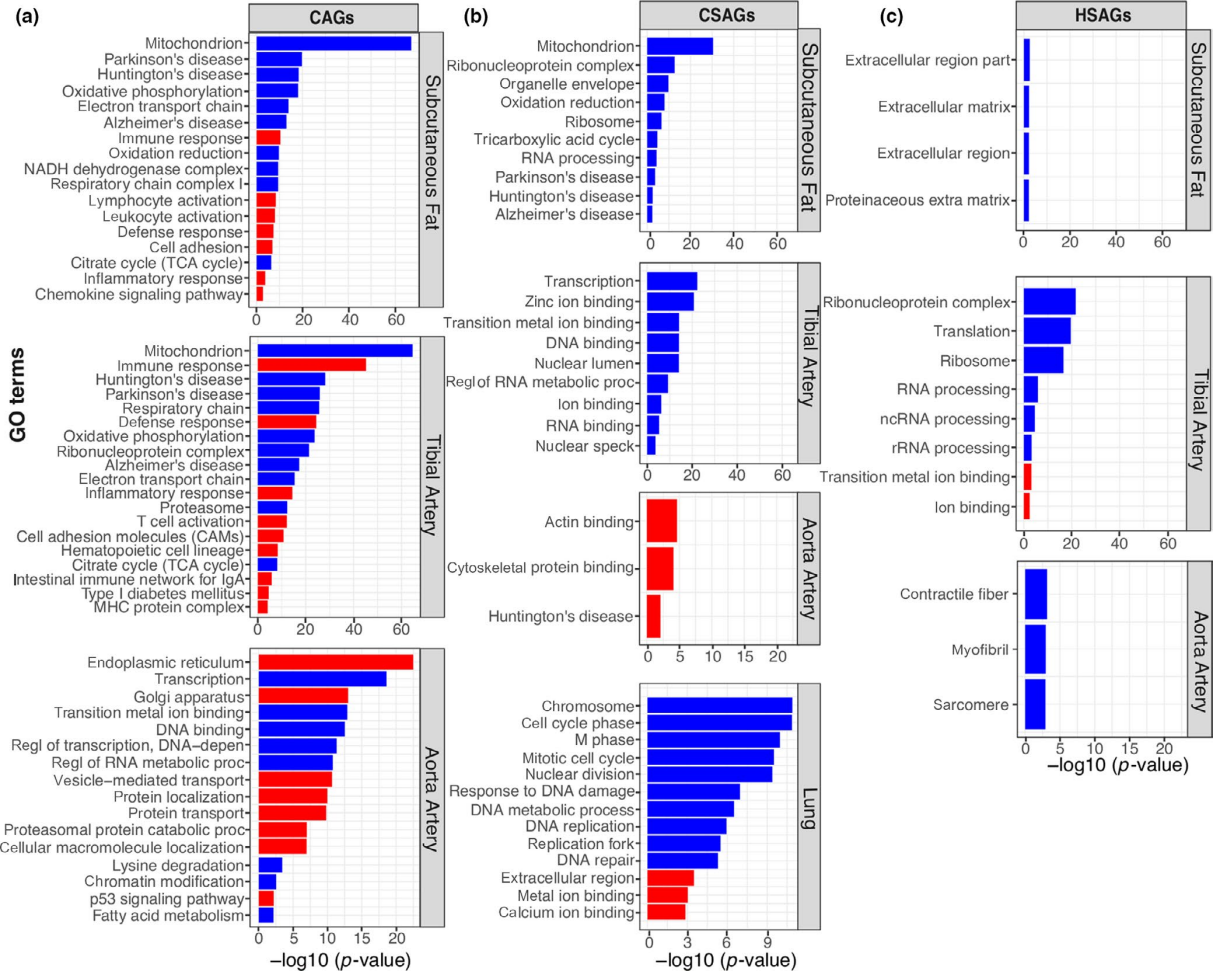
Function enrichment of the CAGs, CSAGs, and HSAGs. (a) GO terms and KEGG pathways enriched in the CAGs. (b) GO terms and KEGG pathways enriched in the CSAGs. (c) GO terms and KEGG pathways enriched in the HSAGs. The red bars denote upregulated genes with age, the blue bars represent downregulated genes with age

GO annotation and pathway analysis revealed differential function enrichment for genes involved in CAGs, CSAGs and HSAGs. Among genes downregulated with age, subcutaneous fat CAGs and CSAGs and tibial artery CAGs were characterized with changes in mitochondrial function, energy/oxidation derivation, and several neurodegenerative diseases. Mitochondria have been found intimately linked to a wide range of processes associated with aging including senescence, inflammation and age‐dependent decline in tissue and organ function (Cui, Kong, & Zhang, 2012; Sun, Youle, & Finkel, 2016). Downregulated HSAGs in tibial artery were found related to ribosome, RNA processing and the regulation of translation. Downregulated CSAGs in lung were found to be involved in cell‐cycle, while no functional enrichments were found in lung HSAGs (Figure 3 and Table S11).

Genes upregulated with age were enriched for different functions compared with genes downregulated with age (Figure 3). For example, CAGs in subcutaneous fat were characterized for response to immune/defense/inflammatory, T‐cell receptor signaling pathway and chemokine signaling pathway. It has been reported that aging is associated with increased T‐cell chemokine expression (Chen et al., 2003). Upregulated CAGs in tibial artery were enriched for similar functions as adipose tissue, and in addition, they were also found associated with intestinal immune network for IgA production and MHC protein complex. Very few functions were observed in upregulated CSAGs and HSAGs, and they were mostly associated with extracellular component (a full list of functional annotations is provided in Table S11).

### 2.8 Link GTEx age‐associated gene expression with known diseases and candidate human aging genes

**Figure 4:**
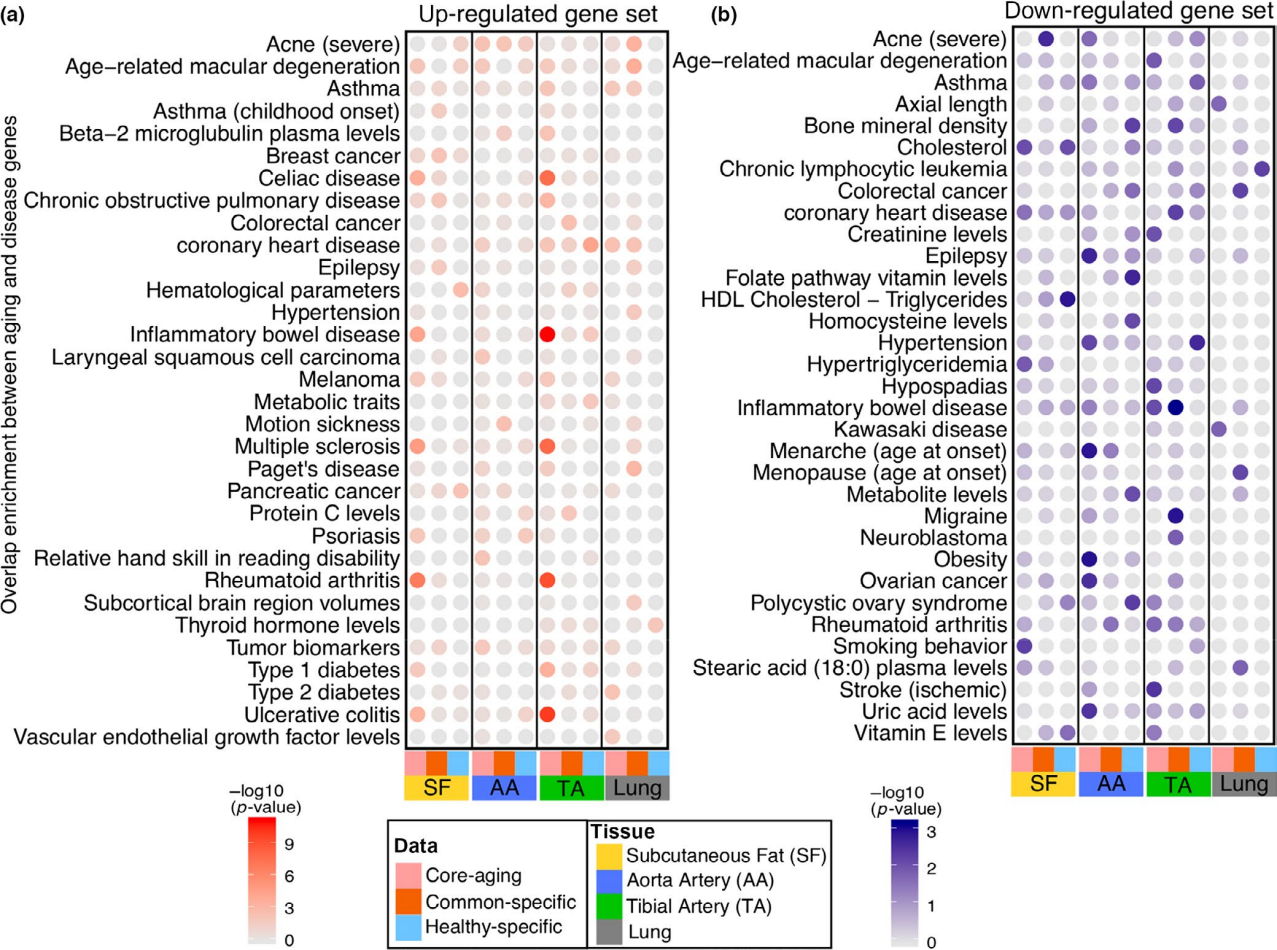
Enrichment of disease genes in three aging gene sets. (a, b) The enrichment between up/downregulated aging genes and complex disease genes in three aging gene sets (from left to right: CAGs, CSAGs or HSAGs) corresponding to four tissues (subcutaneous fat (SF); aorta artery (AA), tibial artery (TA), and Lung). −log_10_ transformed *p*‐values were displayed in a color‐scale with more solid colors corresponding to more significant *p*‐values

Previously, we compiled a list of disease genes for 277 diseases/traits based on the NIH Genome‐wide association study (GWAS) catalog (Welter et al., 2014) and Online Mendelian Inheritance in Man (OMIM) (Amberger, Bocchini, Schiettecatte, Scott, & Hamosh, 2015)(see Methods for details). Using this combined gene list, we investigated the disease gene enrichment for CAGs, CSAGs, and HSAGs in four tissues, considering up‐/downregulated aging genes separately. To visualize the results, we displayed the top 5 disease/traits that had significant enrichment in at least one type of aging genes for each tissue in Figure 4.

We found that disease gene enrichment for “healthy” and “common” aging signatures varied in a tissue‐specific manner. For the upregulated aging genes, most of the significant disease enrichment was observed in either CAGs or CSAGs but to a lesser degree in the HSAGs (Figure 4a). For example, in tibial artery, CAGs were found to be strongly associated with multiple bowel diseases, including ulcerative colitis, inflammatory bowel disease and celiac disease, but very few enrichments were observed for the HSAGs.

For the downregulated aging genes, we generally observed more significant overlaps with various disease traits for all types of aging genes, including the HSAGs (Figure 4b). For example, CAGs in subcutaneous fat were overrepresented for disease traits like cholesterol and hypertriglyceridemia; while downregulated HSAGs were strongly related to HDL cholesterol‐triglycerides (“good” cholesterol). HSAGs also showed strong enrichment for genes related to ribosome and RNA processing (Figure 3c); previous studies have reported that both caloric restriction and rapamycin treatment extend health/lifespan and substantially decrease mRNA levels of ribosomal proteins through reduced mTOR activity (Frenk & Houseley, 2018); therefore, regulation of the ribosomal proteins could bring benefit to the healthy tissue aging. We also performed gene set enrichment analysis (GSEA) to the age‐associated genes in subcutaneous fat and found they were enriched for very few diseases (FDR < 0.05) in either the “common” aging or “healthy” aging cohorts (Figure S6).

Last but not least, we tested the enrichment of literature curated candidate human aging genes with respect to “healthy” and “common” aging signatures. A total of 307 candidate human aging genes were downloaded from GenAge (de Magalhaes & Toussaint, 2004). We then calculated the overlap enrichment with three aging gene sets (Table S12). Our results showed that CAGs in subcutaneous fat and aorta artery were enriched for human GenAge genes (*p*‐value < .01), neither CSAGs nor HSAGs were enriched for them. However, in lung, CSAGs have stronger overlap with GenAge genes compared to CAGs and HSAGs.

## 3 DISCUSSION

We studied the difference between GTEx “healthy” and “common” aging at a transcriptome level by leveraging GTEx data. GTEx “healthy” and “common” aging shared a large proportion of genes, suggesting the existence of a core aging program regardless of the individual's health status. Despite the large overlap between “healthy” and “common” aging genes, HSAGs and CSAGs showed different function enrichment, and CAGs/CSAGs had higher enrichment for disease genes. Since certain CSAGs become age‐associated only when disease individuals are included, their association with age in the “common” cohort is therefore likely driven by the disease. We also noticed that most of the age‐associated gene expression changes were relatively small based on the log fold change, this indicate that the difference between “healthy” and “common” aging was likely a result of the accumulation of small gene expression difference in hundreds to thousands of genes.

Disease‐associated gene regulations are overall different from age‐associated genes, supporting that aging and disease are fundamentally distinct in their gene regulations. However, disease‐associated transcriptome signatures do share some common genes with “healthy” aging signatures. For these shared genes, the direction of gene regulation in “healthy” aging is largely consistent with the regulation direction induced by disease. This suggests that transcriptome regulation in healthy aging could facilitate the development of disease. For example, even in the “healthy” aging adipose tissues, we observed elevated inflammation gene expression (e.g., *CDKN2A*, *IL4R*, *TGFB1,* and *PTPN22* in CAGs and *TNFS4F* in the HSAGs), and it has been noticed that obesity‐related chronic low‐grade inflammation is responsible for the decrease of insulin sensitivity (L. Chen, Chen, Wang, & Liang, 2015).

We speculated that some “healthy‐specific” aging genes may provide protective mechanisms to prevent disease development, therefore to promote a healthy aging phenotype. Among the top few upregulated HSAGs in the subcutaneous fat were *KLF4*, *EAF2,* and *ABCA8* (S2_Data). The overexpression of *ABCA8* can lead to significant increase of plasma HDLc levels (Trigueros‐Motos et al., 2017). The *EAF2* gene has complex and overall protective functions in different cell and tissue types. For example, *EAF2* is a key factor mediating androgen protection of DNA damage via Ku70/Ku80 in prostate cancer cells (Ai et al., 2017). It may also suppress oxidative stress‐induced apoptosis of HLE‐B3 cells exerted through the activation of Wnt3a signaling (Feng & Guo, 2018). *KLF4* functions as an immediate‐early regulator of adipogenesis specifically induced in response to cAMP (Birsoy, Chen, & Friedman, 2008), while abiogenesis is known to be reduced in elderly individuals and correlates with the deteriorated functions of old adipose tissues (Kirkland, Tchkonia, Pirtskhalava, Han, & Karagiannides, 2002). It could be an important and unique feature for the healthy aging program to regulate these protective genes to provide the resilience in these aging tissues.

Accumulating evidence has shown the impact of sex dimorphism on aging and gene expression across mammal tissues (Naqvi et al., 2019; Sampathkumar et al., 2019). For example, in both the “common” and “healthy” cohorts of subcutaneous fat, we found *CDKN2A* in males showed a stronger upregulation with age compared to females (the coefficient of sex term was significantly nonzero with FDR = 2.05 × 10^−9^ and 1.21 × 10^−8^ in the “common” and “healthy” cohorts, respectively) (Figure S7). Loss of *CDKN2A* has been found to induce sexually dimorphic leanness in female mice (Kim et al., 2019). Systematically understanding the underlying transcriptional impact on sex differences in aging will be crucial to tailor therapeutic strategies that target sex‐specific disease mechanisms.

We pointed out that our definition of GTEx “healthy” aging cohort is different from a strict‐sense healthy aging population. Since our “healthy” aging is defined at tissue level, this does not exclude the possible cross‐talk between certain disease categories with the tissue type under consideration. For instance, the “healthy” cohort for subcutaneous fat could contain individuals with ischemic heart disease, asthma or chronic obstructive pulmonary disease (COPD). Whether this approach can reliably recapitulate the relationship between healthy aging and common aging need to be verified in the future. In addition, we used all GTEx samples to approximate the general aging population in the society (“common” aging). Further investigation is needed to evaluate if the GTEx cohort can represent the general aging population. On the other hand, it has been and will continue to be difficult to collect essential tissues from truly healthy individuals. Therefore, we consider this work can serve as an intermediate step to understanding healthy aging in the strict‐sense.

In conclusion, we performed a comparative analysis of “healthy” and “common” aging genes based on transcriptomic data from GTEx. We found “common” aging signatures are comparably more associated with genes and pathways that cause disorders during aging process, while “healthy” aging is likely to contain genes and pathways that boost resilience. As a future direction, a meaningful effort would be to catalog the protective aging gene regulations in details and identify actionable targets to promote the healthy aging program in “common” aging populations.

## 4 METHODS

### 4.1 Linear regression model for age‐associated gene detection

We implemented a linear regression model to identify age‐associated gene expression (Equation 1).

(1) $$Y_{\mathrm{ij}}=\beta_{j}+\gamma_{j}{\text{Age}}_{i}+\delta_{j}{\text{Sex}}_{i}+\sum_{k=1}^{3}\mu_{\mathrm{jk}}{\text{Genotype}}_{\mathrm{ik}}+\sum_{k=1}^{N}\alpha_{\mathrm{jk}}{\text{PC}}_{\mathrm{ik}}+\theta_{j}{\text{RIN}}_{i}+\delta_{j}{\text{PMI}}_{i}+\varepsilon_{\mathrm{ij}}.$$

More details of the model are provided in Text S3.

### 4.2 Differential expression between the “disease” and “healthy” individuals

For differential expression analysis, we used the statistical methods implemented in the limma‐voom package (Table S6). We created a design matrix taking into account 2 conditions (i.e., “disease” and “healthy” cohorts) and considered several covariates:

$$\text{Design}=\text{model}.\text{matrix}\left(∼\text{condition}+\text{GENDER}+\text{AGE}+\text{SMRIN}+\text{SMTSISCH}+\sum_{k=1}^{3}\mu_{\mathrm{jk}}{\text{Genotype}}_{\mathrm{ik}}\right).$$

FDR value < 0.05 was considered as significant DEGs. Additional information can be found in Text S4.

### 4.3 Function annotation for GTEx aging signatures

DAVID tool was used to perform GO annotation. CAGs, CSAGs, and HSAGs gene lists were submitted to DAVID by choosing GO_FAT and KEGG pathway terms to describe the overrepresented functional terms. The threshold for overrepresented GO terms was set to FDR < 0.05.

### 4.4 Assemble disease gene list and identify significant overlap between disease and aging genes

Disease genes were retrieved from two sources: NIH GWAS Catalog and OMIM. We only considered genes in the GWAS catalog with *p*‐value < 5 × 10^−8^, a widely accepted threshold for genome‐wide significance. Clustering and manual curation were used to merge genes in GWAS and OMIM. We only considered disease categories that contained with at least five genes. We then performed a Hypergeometric based test between the disease genes and three age‐associated gene sets in four tissues. Fast gene set enrichment analysis (fgsea) (Sergushichev, 2016) was used to carry out the GSEA in subcutaneous fat, which is an R friendly package that generates equivalent results as the GSEA web tool from Broad Institute.

## CONFLICT OF INTEREST

None declared.

## AUTHOR CONTRIBUTIONS

Z.T. conceived and designed the project; L.Z. performed the analysis; L.Z. and Z.T. wrote the paper; J.Y., S.P., J.Z., B.Z., and Y.S. contributed to the discussion of the results and helped revise the paper. All authors reviewed and approved the manuscript.

## Data Availability

GTEx V7/V8 data were downloaded from the Database of Genotypes and Phenotypes (dbGaP) under accession phs000424.v7.p2 and phs000424.v8.p2 (Table S1). Details about the data processing are provided in the Text S2.
