# Supplementary material for: Transcriptome analysis reveals the difference between “healthy” and “common” aging and their connection with age‐related diseases
Source: Aging Cell. 2020 Feb 19;19(3):e13121. doi: 10.1111/acel.13121 (PMC7059150; doi:10.1111/acel.13121)
Supplement: Supplementary file 1 [file ACEL-19-e13121-s001.docx]

**Supplemental Text**

**S1 Determining the “healthy” cohort**

In order to identify “healthy” *vs.* less “healthy” tissues, we explored different strategies to evaluate which GTEx “healthy” aging cohort might better approximates the strict healthy aging cohort. We stratified a “healthy” cohort by excluding individuals annotated with one or more GTEx documented diseases (e.g., type 2 diabetes, cardiovascular disease, stroke, chronic obstructive pulmonary disease (COPD)), which we called it “disease-free” cohort as some of the remaining individuals may still not be truly healthy. A large number of samples were filtered out due to this procedure applied to **GTEx v8 data** (e.g., only 94 out of 581 samples remained in subcutaneous fat), which will significantly reduce the statistic power to identify age-associated genes. An alternative strategy to study healthy aging is at the tissue level, we removed samples whose donors were annotated with diseases biologically relevant to that tissue. Use subcutaneous fat as an example, we defined a “tissue-level healthy” cohort formed by donors without type 2 diabetes and BMI < 30. We constructed two disease cohorts to compare with the “healthy” cohorts: 1. a “disease” cohort that contains any kind of the GTEx documented disease, which is basically all donors excluding “disease-free” donors; 2. a “tissue-level disease” cohort that contains donors with type 2 diabetes and BMI >=30 (Table T1) for the adipose tissue. We then used limma to identify GTEx DEGs between “disease-free” cohort *vs.* “disease” cohort; “disease-free” cohort *vs.* “tissue-level disease” cohort; “tissue-level healthy” cohort *vs.* “tissue-level disease” cohort. We also compared these two approaches with prior independent studies to decide which approach may give a better approximation to healthy aging.

Our results showed that 1) in the “disease-free” cohort, we identified much fewer age-associated genes (Table T1), however, “healthy” and “common” aging still shared a large number of gene regulations (Figure T1); 2) DEG analysis between “disease-free” vs. “disease” cohort (Table T2) showed no significant enrichment in disease-related DEGs, this is possibly because the “disease” cohort may contain many relatively healthy samples for that tissue (e.g., a COPD patient could still have a relatively healthy adipose tissue). When the comparison was made between “disease-free” cohort with “tissue-level disease” samples, we do see the DEGs significantly overlap with disease-related DEGs. When considering the “tissue-level healthy” cohort, GTEx DEGs identified from subcutaneous fat were most significantly enriched in the obesity-related, and insulin-resistance DEGs (Table T2). We consider this is likely due to the increased statistical power as the sample size of “tissue-level healthy” samples are much larger than the sample size of “disease-free” cohort.

Based on this result, the limited power due to the small sample size of the “disease-free” cohort, and the apparent biased age-distribution for GTEx “disease-free” cohort (relatively more samples in the younger ages compared to “unhealthy” cohort), we consider investigating the GTEx “healthy” aging at the tissue level.

**Table T1.** **Different definition of aging cohorts, their sample size and number of identified age-associated genes.**

| Subcutaneous Fat | all | “disease-free” | “disease | “tissue-level healthy” | “tissue-level disease” |
| --- | --- | --- | --- | --- | --- |
| No. of samples | 581 | 94 | 487 | 328 | 253 |
| No. of aging gene | 5461 | 1046 | 1456 | 4124 | 58 |

**Table T2.** **Comparison of disease-related DEGs derived from different GTEx “healthy” cohort definitions with disease DEGs from previous independent studies.**

We compared disease-related DEGs from GTEx with prior independent studies (obesity, insulin resistance, CHD and COPD) in subcutaneous fat. No. of background genes refers to the number of genes used for DEG analysis in subcutaneous fat; No. of identified DEGs is the number of disease DEGs generated from GTEx; No. of reported disease genes shows the number of disease-related genes from previous studies that overlapped with our background genes in subcutaneous fat; No. of overlapped aging genes is the number of overlapped genes between GTEx and prior study; p-value was calculated by using hypergeometric test.

| Subcutaneous Fat |  | Obesity | Insulin resistance | CHD | COPD |
| --- | --- | --- | --- | --- | --- |
| “disease-free” *vs.* “disease” | No. of background genes | **21530** | | | |
|  | No. of identified DEG genes | **18** | | | |
|  | No. of reported disease genes | 38 | 140 | 30 | 78 |
|  | No. of overlapped DEG genes | 0 | 0 | 0 | 0 |
|  | p-value of hypergeometric test | 1 | 1 | 1 | 1 |
| “disease-free” *vs.* “tissue-level disease” | No. of background genes | **20973** | | | |
|  | No. of identified DEG genes | **672** | | | |
|  | No. of reported disease genes | 38 | 140 | 30 | 78 |
|  | No. of overlapped DEG genes | 4 | 17 | 0 | 0 |
|  | p-value of hypergeometric test | 0.03 | 2.57e-06 | 1 | 1 |
| “tissue-level healthy” *vs*. “tissue-level disease” | No. of background genes | **21532** | | | |
|  | No. of identified DEG genes | **2594** | | | |
|  | No. of reported disease genes | 38 | 140 | 30 | 78 |
|  | No. of overlapped DEG genes | 12 | 84 | 4 | 10 |
|  | p-value of hypergeometric test | 1.2e-03 | 1.10e-41 | 0.50 | 0.47 |

**Fig. T1.** **The relationship among the age-associated genes from the “common” cohort, and age-associated genes from the “disease-free” cohort in subcutaneous fat.** 581 samples were used to identify age-associated genes from the “common” cohort, and 94 samples were used to calculate age-associated genes from the “disease-free” cohort. 977 age-associated genes overlapped between “common” cohort and “disease-free” cohort.


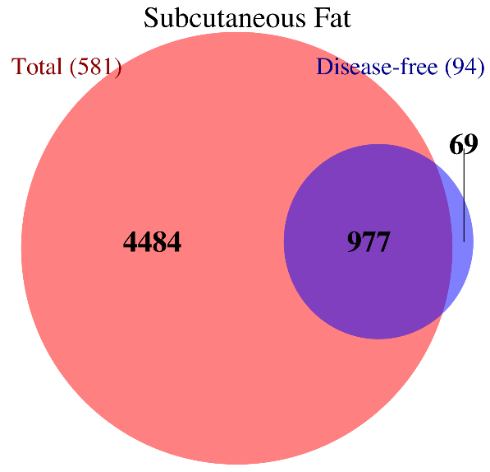


**S2 Data filters**

Form the 54 available tissues in the V7, we started by selecting those with at least 80 samples, and samples with more than 20 million mapping reads and greater than 40% mapping rate. Cell line data were removed from our analysis. Only genes with expression > 0.1 TPM and aligned read count of 5 or more in more than 80% of all samples within each tissue were used for aging gene identification. Expression measurements for each gene in each tissue were subsequently inverse quantile normalized to the quantiles of the standard normal distribution, this helps to reduce the impact of extreme/outlier gene expression values, which could affect the regression fitting.

Our final dataset included samples from 46 tissue types. The sample size in each tissue ranged from 85 to 491, with an average of 247 samples (Fig. S1&S2).

**S3 Details of linear regression**

In our linear regression model, $Y_{ij}$ is the expression level of gene j in sample i, $Age_{i}$ denotes the donor age of sample i, $Sex_{i}$ denotes the sex of donor for sample i, $Genotype_{ik}$($k\in(1,2,3)$) denotes the value of k-th principal component value of genotype profile for the i-th sample, ${PC}_{ik}$($k\in(1,\ldots, N$) denotes the value of k-th principal component value of gene expression profile for the i-th sample, N is the total number of top PCs under consideration, $RIN_{i}$ denotes the RIN score of sample i, $PMI_{i}$ denotes the PMI of sample i, ${}_{ij}$ is the error term, ${}_{j}$, ${}_{j}$, ${}_{jk}$, ${}_{j}$, is the regression coefficient for each variates. The corresponding correlation and p-values (adjusted with BH (Benjamini & Hochberg, 1995) method) were then calculated for all genes; only FDR value < 0.01 were considered as significant age-associated genes. Several covariates (such as genotype PCs and PEER factors) we adjusted in the regression model were selected following the method used by GTEx consortium (Consortium et al., 2017). From consortium’s analysis, the top three genotype PCs were considered sufficient to capture the major population structure in the GTEx dataset and was used for the consortium paper. Since the correction of PEER factors or PCs may also eliminate the age-associated signals if they correlated with age, we explored different gene expression PC combinations to identify the combination generating the greatest number of aging genes. The performance of linear regression metrics (r-squared and mean squared errors) is provided in Fig. S8.

**S4 Details of differential expression analysis**

In our differential expression analysis, condition and gender were converted as factors, type of nucleic acid isolation batch (SMNABTCHT) was implemented as batch. The corresponding correlation and p-values (adjusted with BH) were then calculated for all genes.

**Supplemental Figures**

**Fig. S1. Number of analyzed samples across different tissues.**

46 tissues with more than 80 samples (mean sample size: 214, min: 81, max: 491) were considered for the current study.

**Fig. S2. Characteristics of the samples and tissues used in this study.**

**a** Distribution of age with tissues ordered by the median value. **b** Distribution of sample PMI values (SMTSISCH, in minutes) with tissues ordered by the median value. **c** Distribution of donor PMI values (TRDNISCH, in minutes) with tissues ordered by median value. **d** Distribution of RIN with tissues ordered by the median value.


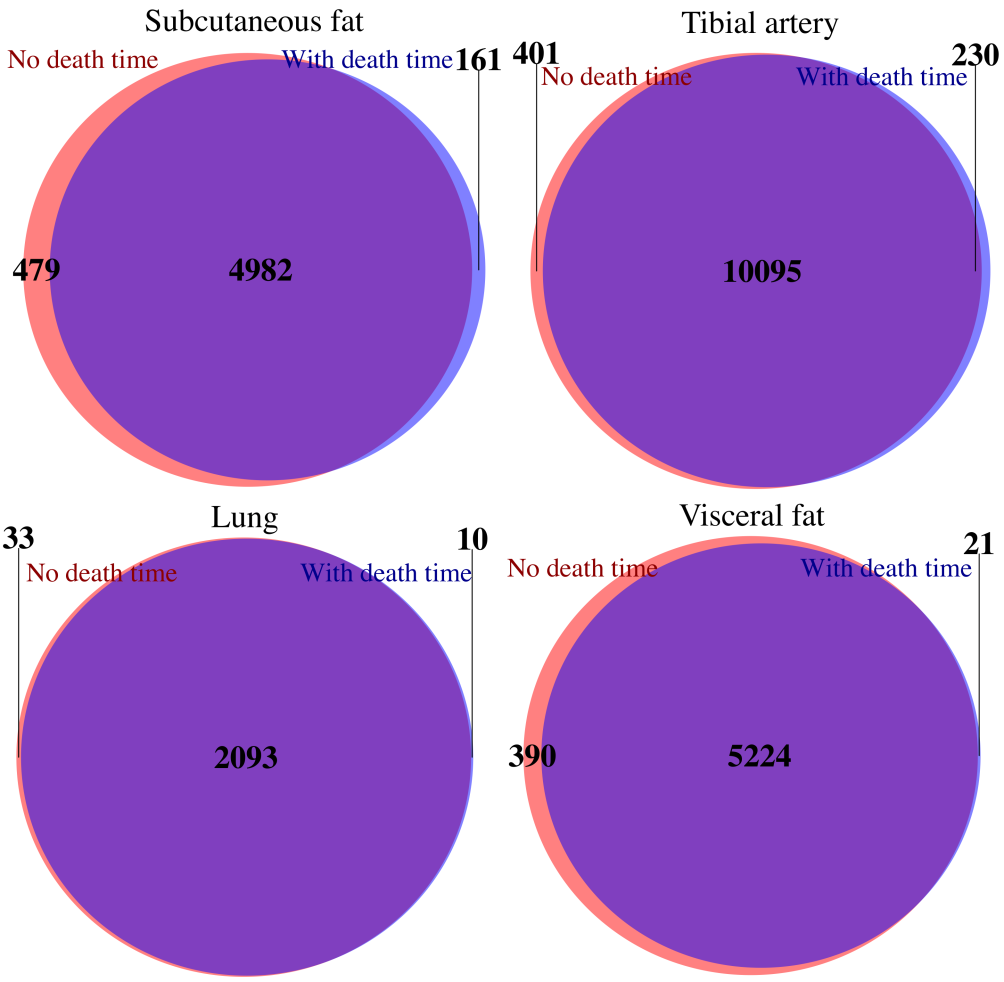


**Fig. S3. Comparison of age-associated genes identified with/without the correction of the death of the time.** No death time defers to age-associated genes identified from our original linear model, with death time represents the death of the time has been added as an extra confounding factor in our original linear model to identify age-associated genes.


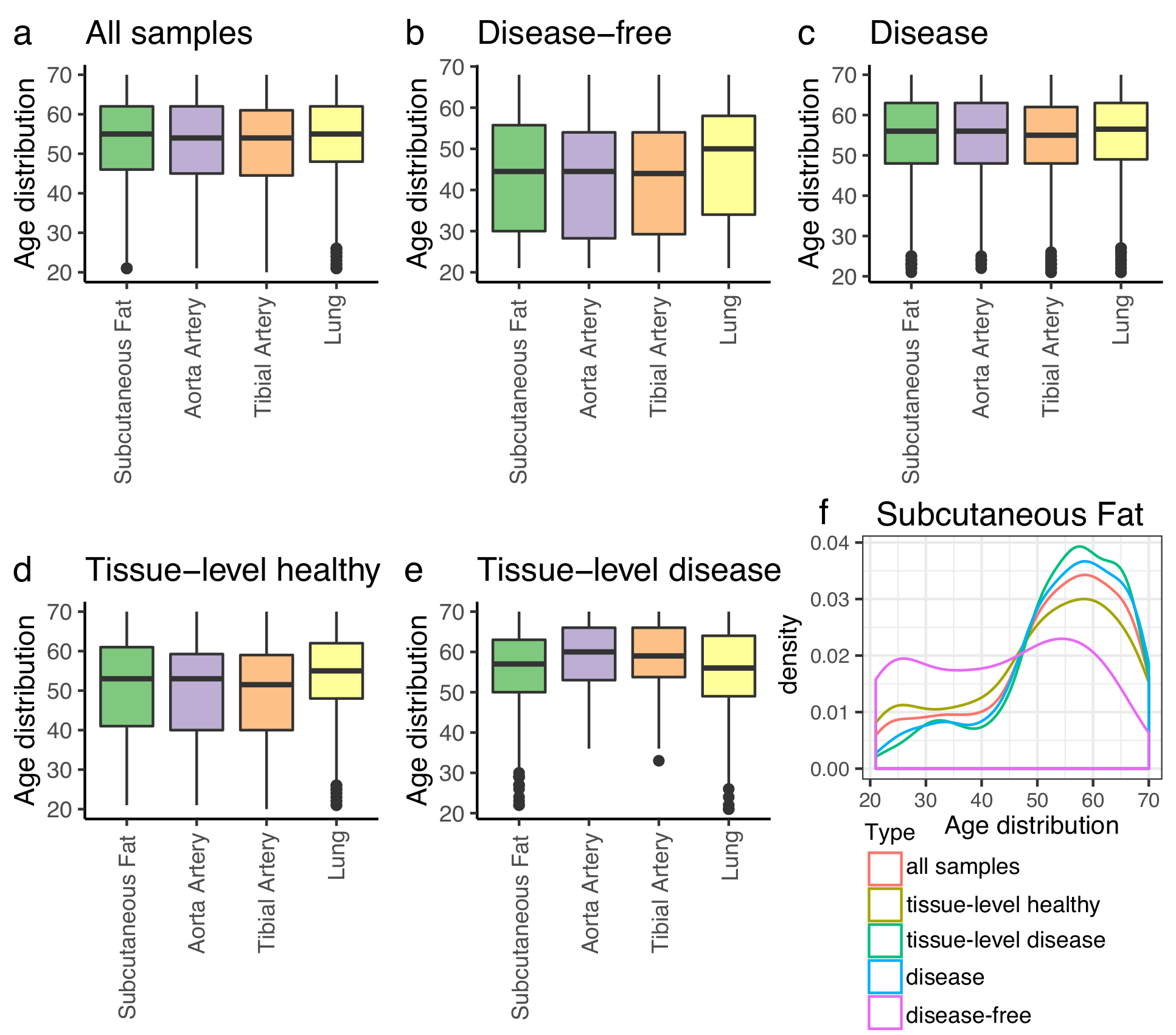


**Fig. S4. Age distribution within different groups in four tissues (subcutaneous fat, aorta artery, tibial artery and lung).**

**a** Distribution of age with tissues ordered by the median value in all samples. **b** Distribution of age with tissues ordered by the median value in disease-free samples. **c** Distribution of age with tissues ordered by the median value in disease samples. **d** Distribution of age with tissues ordered by the median value in tissue-level healthy samples. **e** Distribution of age with tissues ordered by the median value in tissue-level disease samples. **f** Density distribution of age in “common” aging, “healthy” aging and “unhealthy” aging in subcutaneous fat.


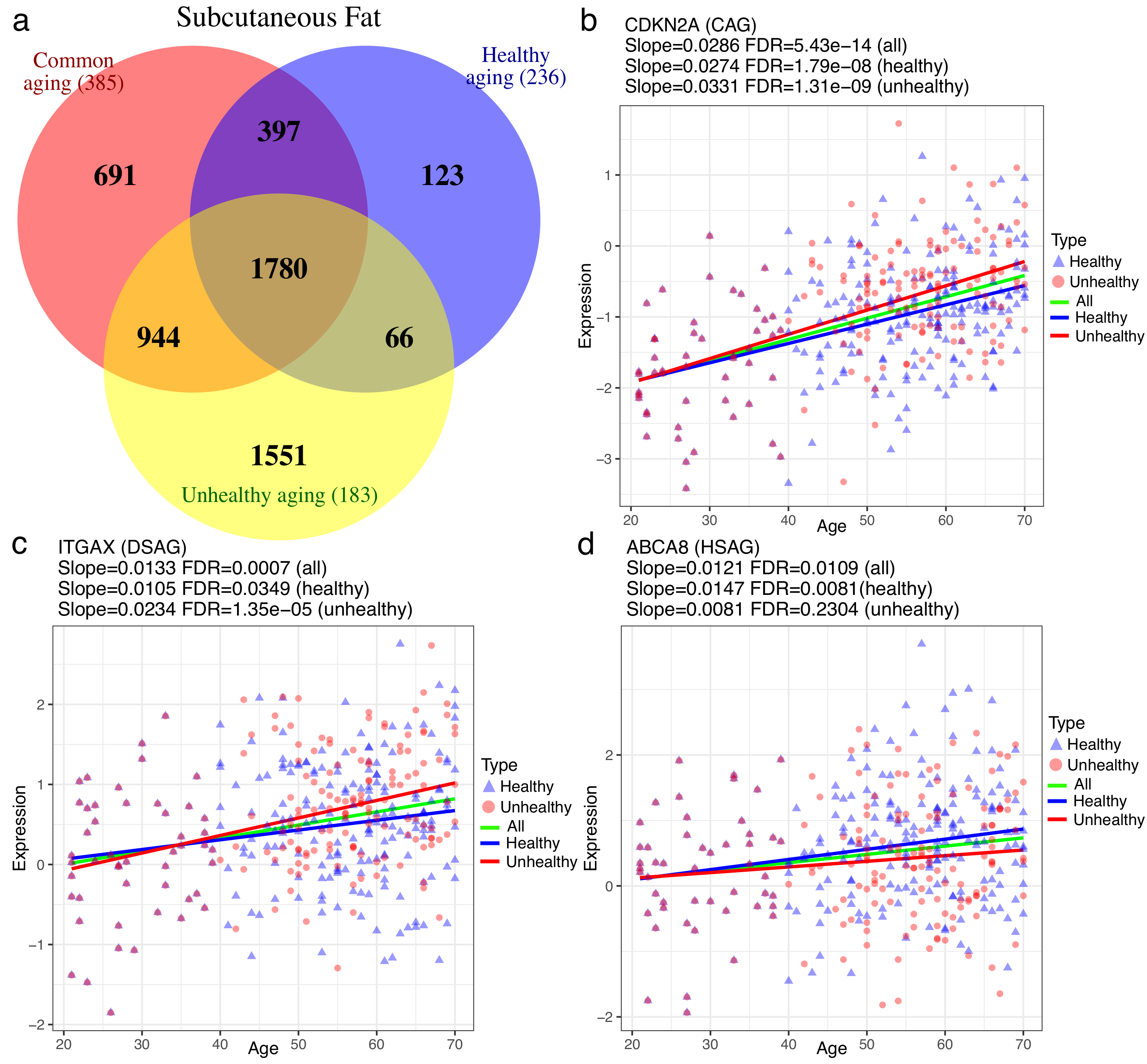


**Fig. S5. Example of age-associated gene expression changes in GTEx subcutaneous fat.** **a** Venn diagram shows the relationship among “common” aging signature identified from the “common” cohort, “healthy” aging signature from the “healthy” cohort, and “unhealthy” aging signature from the “unhealthy” cohort in subcutaneous fat. **b,c,d** Scatter plots show 3 representative age-associated gene expression patterns in three gene sets (from top to bottom: CAGs, CSAGs, or HSAGs). The green line is the regression line from the “common” cohort, the red line and red dots denote the regression line and samples from the “disease” cohort, the blue line and blue dots represent the regression line and samples from the “healthy” cohort.

**Fig. S6. Gene set enrichment of disease genes in subcutaneous fat.**

**a,b** Gene Set Enrichment showing “common”/“healthy” aging signatures evaluated in the context of gene sets representative for complex diseases/traits in subcutaneous fat.


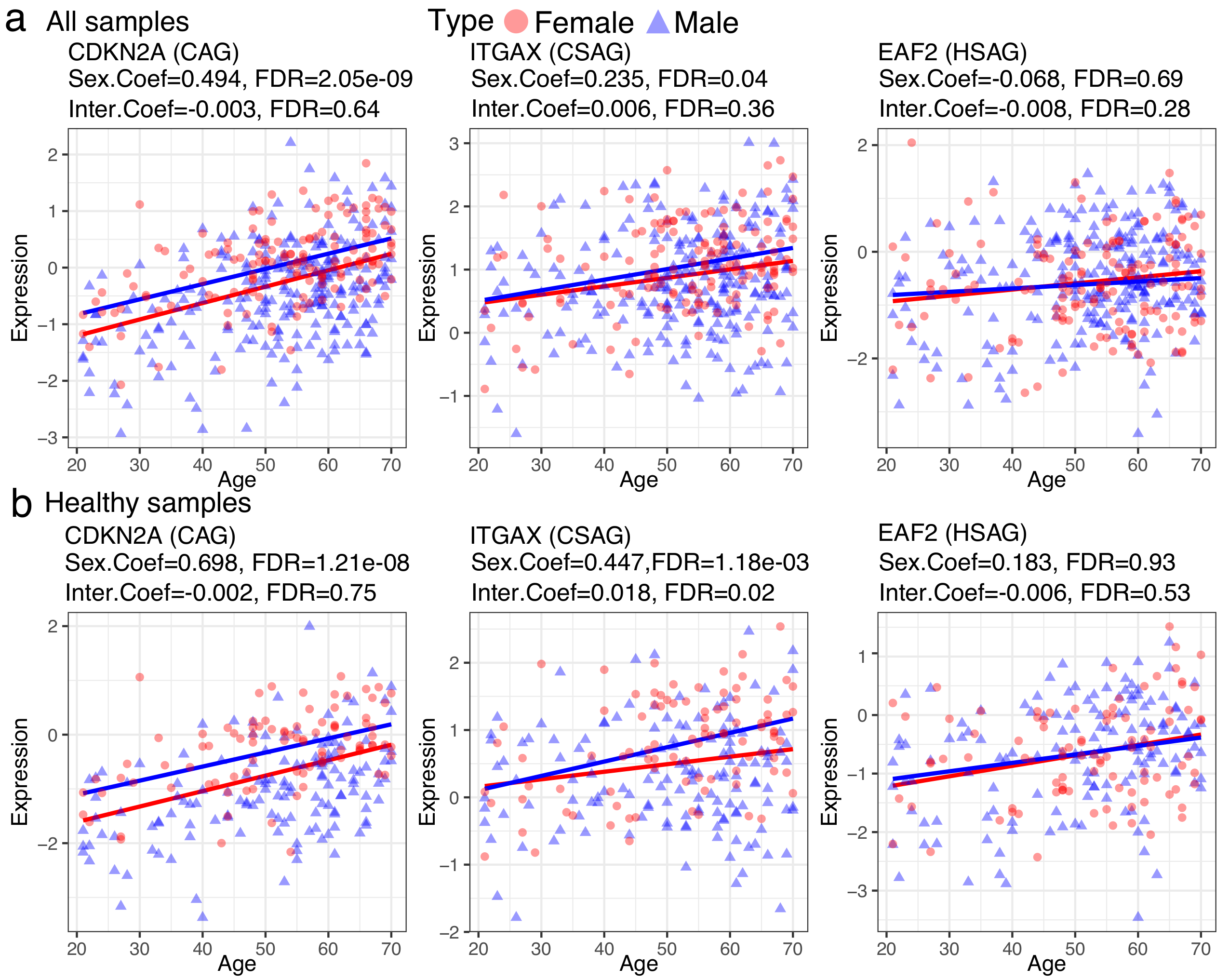


**Fig. S7. Age-associated gene expression changes in subcutaneous fat considering sex and interaction between sex and age.**

**a** Scatterplot of 3 representative age-associated gene expression patterns *CDKN2A*, *ITGAX* and *EAF2* in adipose tissue. Pearson-R value and p-value in the title represents the Pearson correlation coefficient between gene expression and sex, as well as interaction coefficient between age and sex across all samples. **b** Scatterplot of 3 representative age-associated gene expression patterns *CDKN2A*, *ITGAX* and *EAF2* in adipose tissue. Pearson-R value and p-value in the title represents the Pearson correlation coefficient between gene expression and sex, as well as interaction coefficient between age and sex across healthy samples. The solid blue triangles plot male samples and solid red circle female samples. Similarly, the blue and red lines denote the regression lines for male and female samples, respectively.

**Fig. S8. Examples of linear regression metrics in subcutaneous fat and tibial artery.** Histograms display the distribution of r-squared values and mean squared error (MSE) values of age-associated genes in subcutaneous fat and tibial artery, and their corresponded mean value and standard deviation (SD).

**Supplemental Tables**

**Table S1. Table files obtained from dbGap corresponding to the data used in this analysis.**

| Information | Table file |
| --- | --- |
| RNA-seq quality metrics from RNA- SeQC | GTEx_Analysis_2016-01-15_v7_RNA-seq_RNA-SeQCv1.1.8_metrics.tsv |
| Gene read count values | GTEx_Analysis_2016-01-15_v7_RNASeQCv1.1.8_gene_reads.gct.gz |
| Gene TPM values | GTEx_Analysis_2016-01-15_v7_RNASeQCv1.1.8_gene_tpm.gct.gz |
| Samples attributes and annotation | GTEx_v7_Annotations_SampleAttributesDS.txt |
| Subject phenotype | GTEx_v7_Annotations_SubjectPhenotypesDS.txt (pht002742.v2.p1) |
| Covariates | GTEx_Analysis_v7_eQTL_covariates.tar.gz |

**Table S2. Statistics for number and percentage of mapped reads grouped by tissue.** Statistics (mean and standard deviation) on the number of mapped reads and ratio between mapped and total number of reads clustered by tissue.

| Tissue | No. of genes | Mean.MappedReads | Sd.MappedReads | Mean.MapRate | Sd.MapRate |
| --- | --- | --- | --- | --- | --- |
| Adipose – Subcutaneous | 18,643 | 78455663 | 21804026 | 0.95 | 0.13 |
| Adipose – Visceral (Omentum) | 18,390 | 77149455 | 15332060 | 0.98 | 0.03 |
| Adrenal Gland | 18,284 | 79117188 | 19217394 | 0.99 | 0.01 |
| Artery – Aorta | 18,189 | 76947511 | 16914851 | 0.99 | 0.01 |
| Artery – Coronary | 18,391 | 79665499 | 29371450 | 0.99 | 0.01 |
| Artery – Tibial | 17,708 | 78200707 | 28300916 | 0.95 | 0.13 |
| Brain – Amygdala | 18,492 | 82111000 | 23117312 | 0.94 | 0.12 |
| Brain – Anterior cingulate cortex (BA24) | 18,972 | 87250223 | 28491358 | 0.96 | 0.13 |
| Brain – Caudate (basal ganglia) | 18,946 | 85818573 | 24929335 | 0.95 | 0.11 |
| Brain – Cerebellar Hemisphere | 19,615 | 86692991 | 23970432 | 0.97 | 0.08 |
| Brain – Cerebellum | 19,978 | 83991284 | 33357587 | 0.97 | 0.05 |
| Brain – Cortex | 19,589 | 81550306 | 19768994 | 0.97 | 0.10 |
| Brain – Frontal Cortex (BA9) | 19,291 | 83046340 | 25491211 | 0.96 | 0.09 |
| Brain – Hippocampus | 18,532 | 81231667 | 22097922 | 0.94 | 0.13 |
| Brain – Hypothalamus | 19,372 | 83865522 | 38328323 | 0.95 | 0.13 |
| Brain – Nucleus accumbens (basal ganglia) | 19,129 | 87384257 | 23648449 | 0.97 | 0.07 |
| Brain – Putamen (basal ganglia) | 18,431 | 83634842 | 25492095 | 0.96 | 0.10 |
| Brain – Spinal cord (cervical c-1) | 18,893 | 81241592 | 22651081 | 0.95 | 0.14 |
| Brain – Substantia nigra | 18,578 | 80140670 | 20899740 | 0.95 | 0.10 |
| Breast – Mammary Tissue | 18,843 | 79067783 | 18694673 | 0.98 | 0.05 |
| Colon – Sigmoid | 18,677 | 79721048 | 18471108 | 0.99 | 0.00 |
| Colon – Transverse | 18,769 | 81791898 | 41404790 | 0.99 | 0.00 |
| Esophagus – Gastroesophageal Junction | 18,405 | 79430027 | 25542694 | 0.99 | 0.00 |
| Esophagus – Mucosa | 18,415 | 77297357 | 18072573 | 0.99 | 0.01 |
| Esophagus – Muscularis | 18,358 | 79916418 | 26343705 | 0.99 | 0.01 |
| Heart – Atrial Appendage | 17,544 | 81195918 | 18075666 | 0.99 | 0.04 |
| Heart – Left Ventricle | 16,074 | 83930643 | 23935391 | 0.94 | 0.14 |
| Liver | 16,723 | 79329539 | 24039737 | 0.99 | 0.01 |
| Lung | 19,540 | 79838055 | 22739430 | 0.95 | 0.12 |
| Minor Salivary Gland | 19,321 | 77966374 | 15437458 | 0.99 | 0.00 |
| Muscle – Skeletal | 15,861 | 81939535 | 22408949 | 0.95 | 0.12 |
| Nerve – Tibial | 19,844 | 78367693 | 22750389 | 0.94 | 0.14 |
| Ovary | 18,999 | 78475834 | 17739438 | 0.99 | 0.00 |
| Pancreas | 17,502 | 78227793 | 17278379 | 0.99 | 0.03 |
| Pituitary | 20,713 | 76694073 | 16324442 | 0.98 | 0.04 |
| Prostate | 19,965 | 77329076 | 20813219 | 0.98 | 0.03 |
| Skin – Not Sun Exposed (Suprapubic) | 19,146 | 77762358 | 19029748 | 0.98 | 0.06 |
| Skin – Sun Exposed (Lower leg) | 19,241 | 77634086 | 20977077 | 0.95 | 0.13 |
| Small Intestine – Terminal Ileum | 19,546 | 80664324 | 19608017 | 0.99 | 0.00 |
| Spleen | 19,775 | 75379707 | 17900150 | 0.99 | 0.00 |
| Stomach | 18,056 | 77278799 | 16008976 | 0.98 | 0.01 |
| Testis | 27,279 | 81257573 | 20368745 | 0.99 | 0.01 |
| Thyroid | 19,974 | 79179823 | 21679341 | 0.95 | 0.13 |
| Uterus | 19,112 | 77853387 | 16250458 | 0.99 | 0.01 |
| Vagina | 19,097 | 75810358 | 17721686 | 0.99 | 0.00 |
| Whole Blood | 14,440 | 83063665 | 24908554 | 0.91 | 0.16 |

**Table S3. Statistics for comparison of age-associated genes from GTEx and previous independent studies.**

Here we performed a comparison of the estimated age-associated genes from GTEx study to prior independent studies in five tissues (whole blood, lung, skin, subcutaneous fat and brain). No. of background genes defers to the number of selected genes we used for aging genes identification in each tissue; No. of reported aging genes is the number of aging genes from previous studies that overlapped with our background genes; No. of GTEx aging genes is the total number of aging genes identified from GTEx; No. of overlapped aging genes is the number of overlapped genes between current study and prior study; % of overlapped aging genes is measured as $\frac{No. of overlapped aging genes}{No. of identified aging genes}$; p-value is calculated by using hypergeometric test.

| Tissue | | No PMI | With PMI |
| --- | --- | --- | --- |
| Blood | No. of background genes | **14,440** | |
|  | No. of reported aging genes | **1,410** | |
|  | No. of GTEx aging genes | 7282 | 1969 |
|  | No. of overlapped aging genes | 767 | 250 |
|  | % of overlapped aging genes | 10.53 | 12.70 |
|  | p-value | 9.33e-03 | 2.95e-06 |
| Lung | No. of background genes | **19,540** | |
|  | No. of reported aging genes | **3,178** | |
|  | No. of GTEx aging genes | 2642 | 980 |
|  | No. of overlapped aging genes | 774 | 363 |
|  | % of overlapped aging genes | 29.30 | 37.04 |
|  | p-value | 2.19e-74 | 4.32e-59 |
| Skin | No. of background genes | **19,451** | |
|  | No. of reported aging genes | **1,323** | |
|  | No. of GTEx aging genes | 3745 | 1067 |
|  | No. of overlapped aging genes | 289 | 124 |
|  | % of overlapped aging genes | 7.72 | 11.62 |
|  | p-value | 7.98E-03 | 2.09e-09 |
| Subcutaneous Fat | No. of background genes | **18,643** | |
|  | No. of reported aging genes | **162** | |
|  | No. of GTEx aging genes | 6142 | 3812 |
|  | No. of overlapped aging genes | 73 | 47 |
|  | % of overlapped aging genes | 1.19 | 1.23 |
|  | p-value | 8.35e-04 | 5.78e-03 |
| Brain | No. of background genes | **22,400** | |
|  | No. of reported aging genes | **4,847** | |
|  | No. of GTEx aging genes | 8134 | 8199 |
|  | No. of overlapped aging genes | 2690 | 2626 |
|  | % of overlapped aging genes | 33.07 | 32.03 |
|  | p-value | 1.59e-209 | 5.81e-176 |

**Table S4.** **Number of age-associated genes identified from GTEx tissues.** 46 tissues were listed in the first column, and their corresponding sample size in the second column. The third column contained the number of age-associated genes when adjusted either by PMI or without. *represents PMI values based on donor ischemic time, otherwise we used sample ischemic time for PMI.

| **Tissue** | **sample size** | **No. of identified age-associated genes** | |
| --- | --- | --- | --- |
|  |  | **-adj PMI^1^** | **+adj PMI^2^** |
| Adipose – Subcutaneous | 385 | 6,142 | 3,812 |
| Adipose – Visceral (Omentum) | 313 | 2,605 | 1,363 |
| Adrenal Gland | 174 | 354 | 366 |
| Artery – Aorta | 267 | 5,902 | 5,826 |
| Artery – Coronary | 151 | 31 | 33 |
| Artery – Tibial | 382 | 8,911 | 8,709 |
| Brain – Amygdala* | 88 | 925 | 1,397 |
| Brain – Anterior cingulate cortex (BA24)* | 109 | 3,359 | 3,277 |
| Brain – Caudate (basal ganglia)* | 142 | 519 | 500 |
| Brain – Cerebellar Hemisphere* | 125 | 495 | 299 |
| Brain – Cerebellum | 154 | 1,158 | 1,005 |
| Brain – Cortex | 136 | 3,729 | 3,545 |
| Brain – Frontal Cortex (BA9)* | 118 | 200 | 164 |
| Brain – Hippocampus* | 111 | 3,135 | 3,296 |
| Brain – Hypothalamus* | 107 | 1,062 | 1,277 |
| Brain – Nucleus accumbens (basal ganglia)* | 130 | 49 | 36 |
| Brain – Putamen (basal ganglia)* | 111 | 21 | 21 |
| Brain – Spinal cord (cervical c-1)* | 81 | 0 | 0 |
| Brain – Substantia nigra* | 80 | 1 | 0 |
| Breast – Mammary Tissue | 250 | 850 | 80 |
| Colon – Sigmoid | 203 | 1,038 | 711 |
| Colon – Transverse | 246 | 181 | 50 |
| Esophagus – Gastroesophageal Junction | 213 | 112 | 164 |
| Esophagus – Mucosa | 358 | 2,547 | 2,303 |
| Esophagus – Muscularis | 335 | 80 | 83 |
| Heart – Atrial Appendage | 264 | 245 | 260 |
| Heart – Left Ventricle | 271 | 114 | 55 |
| Liver | 153 | 7 | 7 |
| Lung | 379 | 2,642 | 980 |
| Minor Salivary Gland | 85 | 172 | 303 |
| Muscle – Skeletal | 491 | 6,672 | 4,444 |
| Nerve – Tibial | 355 | 5,779 | 3,619 |
| Ovary | 122 | 2,615 | 1,809 |
| Pancreas | 220 | 48 | 44 |
| Pituitary | 157 | 3 | 1 |
| Prostate | 132 | 913 | 1,556 |
| Skin – Not Sun Exposed (Suprapubic) | 333 | 2,317 | 319 |
| Skin – Sun Exposed (Lower leg) | 410 | 2,017 | 823 |
| Small Intestine – Terminal Ileum | 122 | 1 | 1 |
| Spleen | 146 | 241 | 196 |
| Stomach | 237 | 275 | 195 |
| Testis | 225 | 1,009 | 639 |
| Thyroid | 397 | 3,307 | 1,150 |
| Uterus | 101 | 2,371 | 2,459 |
| Vagina | 106 | 66 | 74 |
| Whole Blood | 369 | 7,282 | 1,969 |

**Table S5. Statistics of age-associated genes overlapped between GTEx brain and previous independent brain studies.**

Here we performed a comparison between age-associated genes from three GTEx brain regions (cortex, cerebellum and hippocampus) to four prior independent studies in brain (Kumar et al.: cerebral cortex and cerebellum; Berchtold et al.: hippocampus, entorhinal cortex, superior-frontal gyrus, and postcentral gyrus; Rhinn et al.: cerebral cortex; Twine et al.: hippocampus and cerebellum.). Table shows the number/percentage/Jaccard Index of overlapped aging genes between each study. The percentage is calculated as: $\frac{A\cap B}{A}$, Jaccard Index is measured as: $\frac{|A\cap B|}{\left| A \right|+\left| B \right|-|A\cap B|}$. For example, A refers to the number of aging genes identified from GTEx Brain (5955), B is the number of aging genes generated from Study 1 (58).

|  | GTEx Brain  (3 regions) | Kumar et al.  (2 regions) | Berchtold et al.  (4 regions) | Rhinn et al.  (1 region) | Twine et al. (2 regions) |
| --- | --- | --- | --- | --- | --- |
| GTEx Brain | 5955 |  |  |  |  |
| Kumar et al. | 27;  0.45%;  0.0045 | 58 |  |  |  |
| Berchtold et al. | 1999;  33.57%;  0.1688 | 27;  46.55%;  0.0034 | 7885 |  |  |
| Rhinn et al. | 448;  7.52%;  0.0664 | 5;  8.62%;  0.0039 | 596;  7.56%;  0.0699 | 1236 |  |
| Twine et al. | 1286;  21.59%;  0.1497 | 13;  22.4%;  0.0033 | 1290;  16.36%;  0.1227 | 300;  24.27%;  0.0617 | 3923 |

**Table S6. Summarization of data used in differential expression analysis.** GTEx donors have been separated into three cohorts based on their medical conditions. The first column shows the tissue we considered in this analysis. The second column is the description of medical conditions based on tissue types (see details below); For example, in subcutaneous fat, the “healthy” cohort is donors that without type II diabetes and their BMI <30; in aorta artery and tibial artery, the “healthy” cohort is donors free from ischemic heart disease, heart attack, acute myocardial infarction and acute coronary syndrome; in lung, the “healthy” cohort is donors without chronic respiratory disease, chronic lower respiratory disease, pneumonia and asthma. The third column contains the number of samples and the number of aging genes identified from the “common” cohort; the fourth column includes the number of samples and the number of aging genes identified from the “healthy” cohort. The fifth column shows the number of samples in the “disease” cohort. The sixth column is the number of overlapped aging genes identified from the “healthy” cohort and the “disease” cohort. The seventh column is the number of disease DEGs detected between the “healthy” cohort and the “disease” cohort.

MHT2D: Diabetes mellitus type II (NIDDM, adult onset diabetes);

MHHRTATT: Heart attack, acute myocardial infarction, acute coronary syndrome;

MHHRTDIS: Ischemic Heart Disease (coronary artery disease (CAD), coronary heart disease, ischemic cardiomyopathy);

MHCOPD: Chronic Respiratory Disease (Chronic Obstructive Pulmonary Syndrome (COPD) OR Chronic Lower Respiratory Disease (CLRD) (chronic bronchitis, emphysema, asthma));

MHCLRD: Chronic Lower Respiratory Disease;

MHASTHMA: Asthma; MHPNMNIA: Pneumonia (acute respiratory infection affecting the lungs).

| Tissues | Medical Condition | **“common” cohort** | | **“healthy” cohort** | | **“disease” cohort** | **No. of overlapped aging genes** | **No. of DEG genes** |
| --- | --- | --- | --- | --- | --- | --- | --- | --- |
|  |  | No. of samples | No. of aging genes | No. of healthy samples | No. of aging genes | No. of disease samples |  |  |
| Subcutaneous  Fat | MHT2D  BMI>=30 | 385 | 3,812 | 236 | 2,366 | 149 | 2,177 | 2,137 |
| Aorta Artery | MHHRTATT  MHHRTDIS | 267 | 5,827 | 198 | 4,711 | 69 | 4,322 | 0 |
| Tibial Artery |  | 382 | 8,709 | 292 | 8,174 | 90 | 5,488 | 436 |
| Lung | MHCOPD MHCLRD MHASTHMA MHPNMNIA | 379 | 980 | 257 | 107 | 122 | 97 | 1,025 |

**Table S7. Statistics for** **comparison of disease-associated genes from GTEx and previous studies.**

Here we performed a comparison of the estimated disease DEGs from GTEx to prior independent studies (obesity, insulin resistance, CHD and CPD) in the corresponding tissues (subcutaneous fat, tibial artery and lung). No. of background genes defers to the number of selected genes we used for differential expression analysis in each tissue; No. of GTEx DEGs is the number of disease DEGs generated from GTEx; No. of reported disease genes shows the number of disease-associated genes from previous studies that overlapped with our background genes in each tissue; No. of overlapped DEGs is the number of overlapped genes between GTEx and prior study; p-value is calculated by using hypergeometric test.

| Tissue |  | Obesity | Insulin resistance | CHD | COPD |
| --- | --- | --- | --- | --- | --- |
| Subcutaneous Fat | No. of background genes | **20,473** | | | |
|  | No. of GTEx DEGs | **2,137** | | | |
|  | No. of reported disease genes | 36 | 142 | 30 | 81 |
|  | No. of overlapped DEGs | 11 | 81 | 5 | 8 |
|  | p-value of hypergeometric test | 7.90e-04 | 1.23e-42 | 0.1984 | 0.6195 |
| Tibial Artery | No. of background genes | **19,463** | | | |
|  | No. of GTEx DEGs | **436** | | | |
|  | No. of reported disease genes | 36 | 141 | 31 | 80 |
|  | No. of overlapped DEGs | 1 | 3 | 3 | 3 |
|  | p-value of hypergeometric test | 0.5580 | 0.6151 | 0.0316 | 0.2665 |
| Lung | No. of background genes | **21,644** | | | |
|  | No. of GTEx DEGs | **1,025** | | | |
|  | No. of reported disease genes | 36 | 141 | 32 | 84 |
|  | No. of overlapped DEGs | 8 | 11 | 0 | 12 |
|  | p-value of hypergeometric test | 2.28e-04 | 0.0716 | 1 | 5.79e-04 |

**Table S8.** **Statistics for comparison of three aging signatures and disease DEGs.**

Here we performed a comparison of the estimated disease DEGs from GTEx to three GTEx aging signatures (“common-specific aging genes”, “core-aging genes”, “healthy-specific” aging genes) of the respective tissues (subcutaneous fat, tibial artery and lung). No. of background genes represents the number of selected genes we used to calculate disease DEGs in each tissue; No. of GTEx DEGs is the number of disease DEGs generated from GTEx; No. of aging genes shows the number of aging signatures in each gene set; No. of overlapped genes is the number of overlapped genes between disease DEG and aging signatures; p-value is calculated by using hypergeometric test.

| Tissue |  | “Common-specific” aging genes | “core-aging genes” | | “healthy-specific” aging genes |
| --- | --- | --- | --- | --- | --- |
| Subcutaneous Fat | No. of background genes | **20,473** | | | |
|  | No. of GTEx DEGs | **2,137** | | | |
|  | No. of aging genes | 1635 | 2177 | 189 | |
|  | No. of overlapped genes | 202 | 400 | 17 | |
|  | p-value of hypergeometric test | 0.0054 | 1.36e-32 | 0.7761 | |
| Tibial Artery | No. of background genes | **19,463** | | | |
|  | No. of GTEx DEGs | **436** | | | |
|  | No. of aging genes | 3221 | 5488 | 2686 | |
|  | No. of overlapped DEG genes | 122 | 116 | 74 | |
|  | p-value of hypergeometric test | 9.73e-10 | 0.7876 | 0.0332 | |
| Lung | No. of background genes | **21,644** | | | |
|  | No. of GTEx DEGs | **1,025** | | | |
|  | No. of aging genes | 96 | 884 | 11 | |
|  | No. of overlapped genes | 6 | 88 | 0 | |
|  | p-value of hypergeometric test | 0.3029 | 3.09e-11 | 1 | |

**Table S9. Number/percentage of overlapped genes between reported disease-associated genes and GTEx aging signatures (“healthy”/“common” aging signatures).**

| Disease DEG | Type | No. of overlapped genes | Total no. of reported DEGs | Overlap percentage |
| --- | --- | --- | --- | --- |
| SF-Insulin | “healthy” aging | 38 | 139 | 27.3381295 |
| SF-Insulin | “common” aging | 50 | 139 | 35.971223 |
| SF-Insulin | DEGs only | 83 | 139 | 59.7122302 |
| SF-Obese | “healthy” aging | 8 | 36 | 22.2222222 |
| SF-Obese | “common” aging | 11 | 36 | 30.5555556 |
| SF-Obese | DEGs only | 24 | 36 | 66.6666667 |
| SF-DEG | “healthy” aging | 417 | 2137 | 19.5133365 |
| SF-DEG | “common” aging | 602 | 2137 | 28.1703322 |
| SF-DEG | DEGs only | 1518 | 2137 | 71.03416 |
| TA-CHD | “healthy” aging | 15 | 34 | 44.1176471 |
| TA-CHD | “common” aging | 15 | 34 | 44.1176471 |
| TA-CHD | DEGs only | 15 | 34 | 44.1176471 |
| TA-DEG | “healthy” aging | 190 | 436 | 43.5779817 |
| TA-DEG | “common” aging | 238 | 436 | 54.587156 |
| TA-DEG | DEGs only | 124 | 436 | 28.440367 |
| Lung-COPD | “healthy” aging | 0 | 86 | 0 |
| Lung-COPD | “common” aging | 4 | 86 | 4.65116279 |
| Lung-COPD | DEGs only | 81 | 86 | 94.1860465 |
| Lung-DEG | “healthy” aging | 6 | 1025 | 0.58536585 |
| Lung-DEG | “common” aging | 94 | 1025 | 9.17073171 |
| Lung-DEG | DEGs only | 931 | 1025 | 90.8292683 |

**Table S10. Number of genes up-/down-regulated with age in “core-aging genes”, “common-specific aging genes” and “healthy-specific aging genes”**.

| Tissue | Common-specific | | Core-aging | | Healthy-specific | |
| --- | --- | --- | --- | --- | --- | --- |
|  | up-regulated | down-regulated | up-regulated | down-regulated | up-regulated | down-regulated |
| Subcutaneous Fat | 793 | 844 | 960 | 1219 | 115 | 76 |
| Aorta Artery | 743 | 764 | 2163 | 2160 | 194 | 197 |
| Tibial Artery | 1257 | 1966 | 2630 | 2860 | 1389 | 1299 |
| Lung | 488 | 398 | 72 | 26 | 9 | 4 |

**Table S11a. GO functional annotation in subcutaneous fat:** The first column shows the original database/resource where the terms orient. The second column shows the enriched terms associated with genes up-/down-regulated with age. The third column shows the Benjamini-Hochberg modified statistic for a p-value. Only selected top enriched terms with a p-value less than 5% are shown. Gene lists were classified into “core-aging genes”, “common-specific aging genes” and “healthy-specific aging genes”.

| **“core-aging genes”** | | |
| --- | --- | --- |
| **Genes decreased expression with age** | | |
| **Category** | **Term** | **Adjusted p-value** |
| CC_FAT | GO:0005739~mitochondrion | 2.84E-68 |
| BP_FAT | GO:0006091~generation of precursor metabolites and energy | 5.88E-25 |
| KEGG_PATHWAY | hsa05012:Parkinson's disease | 1.65E-20 |
| KEGG_PATHWAY | hsa05016:Huntington's disease | 4.28E-19 |
| KEGG_PATHWAY | hsa00190:Oxidative phosphorylation | 7.48E-19 |
| BP_FAT | GO:0006119~oxidative phosphorylation | 8.51E-17 |
| BP_FAT | GO:0015980~energy derivation by oxidation of organic compounds | 1.60E-16 |
| BP_FAT | GO:0022900~electron transport chain | 1.23E-14 |
| KEGG_PATHWAY | hsa05010:Alzheimer's disease | 9.66E-14 |
| BP_FAT | GO:0042773~ATP synthesis coupled electron transport | 9.11E-13 |
| BP_FAT | GO:0055114~oxidation reduction | 1.89E-10 |
| CC_FAT | GO:0030964~NADH dehydrogenase complex | 3.85E-10 |
| CC_FAT | GO:0045271~respiratory chain complex I | 3.85E-10 |
| BP_FAT | GO:0006732~coenzyme metabolic process | 4.44E-09 |
| CC_FAT | GO:0005840~ribosome | 1.12E-07 |
| KEGG_PATHWAY | hsa00020:Citrate cycle (TCA cycle) | 3.95E-07 |
| BP_FAT | GO:0009060~aerobic respiration | 9.08E-06 |
| BP_FAT | GO:0006396~RNA processing | 4.77E-05 |
| KEGG_PATHWAY | hsa00620:Pyruvate metabolism | 1.03E-04 |
| **Genes increased expression with age** | | |
| **Category** | **Term** | **Adjusted p-value** |
| BP_FAT | GO:0006955~immune response | 4.03E-11 |
| BP_FAT | GO:0042110~T cell activation | 8.38E-11 |
| BP_FAT | GO:0046649~lymphocyte activation | 4.21E-09 |
| BP_FAT | GO:0006952~defense response | 4.38E-08 |
| BP_FAT | GO:0007155~cell adhesion | 1.27E-07 |
| MF_FAT | GO:0030695~GTPase regulator activity | 4.92E-06 |
| BP_FAT | GO:0045061~thymic T cell selection | 1.54E-06 |
| MF_FAT | GO:0060589~nucleoside-triphosphatase activity | 4.91E-06 |
| MF_FAT | GO:0005096~GTPase activator activity | 2.49E-05 |
| KEGG_PATHWAY | hsa04660:T cell receptor signaling pathway | 2.50E-04 |
| BP_FAT | GO:0006954~inflammatory response | 1.68E-04 |
| BP_FAT | GO:0050851~antigen receptor-mediated signaling pathway | 5.39E-04 |
| MF_FAT | GO:0005509~calcium ion binding | 0.0013 |
| KEGG_PATHWAY | hsa04062:Chemokine signaling pathway | 0.0017 |
| BP_FAT | GO:0030097~hemopoiesis | 0.0020 |
| KEGG_PATHWAY | hsa04666:Fc gamma R-mediated phagocytosis | 0.0143 |
| KEGG_PATHWAY | hsa05340:Primary immunodeficiency | 0.0109 |
| **“common-specific aging genes”** | | |
| **Genes decreased expression with age** | | |
| **Category** | **Term** | **Adjusted p-value** |
| CC_FAT | GO:0005739~mitochondrion | 3.35E-31 |
| BP_FAT | GO:0006091~generation of precursor metabolites and energy | 2.49E-10 |
| CC_FAT | GO:0000502~proteasome complex | 4.40E-11 |
| BP_FAT | GO:0051443~positive regulation of ubiquitin-protein ligase activity | 6.41E-10 |
| BP_FAT | GO:0051351~positive regulation of ligase activity | 1.07E-09 |
| CC_FAT | GO:0031967~organelle envelope | 1.54E-10 |
| KEGG_PATHWAY | hsa03050:Proteasome | 1.94E-07 |
| GOTERM_BP_FAT | GO:0015980~energy derivation by oxidation of organic compounds | 2.41E-07 |
| BP_FAT | GO:0055114~oxidation reduction | 9.78E-09 |
| CC_FAT | GO:0005840~ribosome | 2.41E-07 |
| BP_FAT | GO:0006099~tricarboxylic acid cycle | 1.91E-05 |
| BP_FAT | GO:0006396~RNA processing | 4.77E-05 |
| KEGG_PATHWAY | hsa05012:Parkinson's disease | 2.11E-04 |
| KEGG_PATHWAY | hsa05016:Huntington's disease | 0.00292 |
| KEGG_PATHWAY | hsa05010:Alzheimer's disease | 0.00440 |
| **Genes up-regulated with age** | | |
| **Category** | **Term** | **Adjusted p-value** |
| CC_FAT | GO:0005815~microtubule organizing center | 0.0383 |
| **“healthy-specific aging genes”** | | |
| **Genes increased expression with age** | | |
| **Category** | **Term** | **Adjusted p-value** |
| CC_FAT | GO:0044421~extracellular region part | 0.0029 |
| CC_FAT | GO:0031012~extracellular matrix | 0.0058 |
| CC_FAT | GO:0005576~extracellular region | 0.0066 |
| CC_FAT | GO:0005578~proteinaceous extracellular matrix | 0.0069 |

**Table S11b. GO functional annotation in tibial artery:** The first column shows the original database/resource where the terms orient. The second column shows the enriched terms associated with genes up-/down-regulated with age. The third column shows the Benjamini-Hochberg modified statistic for a p-value. Only selected top enriched terms with a p-value less than 5% are shown. Gene lists were classified into “core-aging genes”, “common-specific aging genes” and “healthy-specific aging genes”.

| **“core-aging genes”** | | |
| --- | --- | --- |
| **Genes decreased expression with age** | | |
| **Category** | **Term** | **Adjusted p-value** |
| CC_FAT | GO:0005739~mitochondrion | 2.45E-65 |
| BP_FAT | GO:0043632~modification-dependent macromolecule catabolic process | 1.06E-31 |
| BP_FAT | GO:0019941~modification-dependent protein catabolic process | 1.06E-31 |
| KEGG_PATHWAY | hsa05016:Huntington's disease | 7.29E-29 |
| KEGG_PATHWAY | hsa05012:Parkinson's disease | 1.36E-26 |
| CC_FAT | GO:0070469~respiratory chain | 2.51E-26 |
| KEGG_PATHWAY | hsa00190:Oxidative phosphorylation | 1.97E-24 |
| BP_FAT | GO:0006091~generation of precursor metabolites and energy | 4.27E-22 |
| CC_FAT | GO:0030529~ribonucleoprotein complex | 4.05E-22 |
| KEGG_PATHWAY | hsa05010:Alzheimer's disease | 5.25E-18 |
| BP_FAT | GO:0022900~electron transport chain | 4.34E-16 |
| CC_FAT | GO:0030964~NADH dehydrogenase complex | 1.86E-16 |
| CC_FAT | GO:0005747~mitochondrial respiratory chain complex I | 1.86E-16 |
| CC_FAT | ribosome | 2.19E-14 |
| BP_FAT | GO:0045184~establishment of protein localization | 1.32E-12 |
| KEGG_PATHWAY | hsa03050:Proteasome | 5.18E-13 |
| BP_FAT | GO:0031398~positive regulation of protein ubiquitination | 1.38E-11 |
| KEGG_PATHWAY | hsa04120:Ubiquitin mediated proteolysis | 2.86E-10 |
| BP_FAT | GO:0008380~RNA splicing | 2.28E-08 |
| KEGG_PATHWAY | hsa00020:Citrate cycle (TCA cycle) | 7.74E-09 |
| MF_FAT | GO:0015078~hydrogen ion transmembrane transporter activity | 1.69E-07 |
| **Genes increased expression with age** | | |
| **Category** | **Term** | **Adjusted p-value** |
| BP_FAT | GO:0006955~immune response | 6.93E-46 |
| CC_FAT | GO:0005886~plasma membrane | 1.57E-32 |
| BP_FAT | GO:0006952~defense response | 3.87E-25 |
| BP_FAT | GO:0009611~response to wounding | 1.69E-17 |
| BP_FAT | GO:0045321~leukocyte activation | 2.93E-16 |
| BP_FAT | GO:0007155~cell adhesion | 4.28E-16 |
| BP_FAT | GO:0006954~inflammatory response | 3.72E-15 |
| BP_FAT | GO:0001775~cell activation | 8.00E-15 |
| BP_FAT | GO:0046649~lymphocyte activation | 3.01E-14 |
| BP_FAT | GO:0042110~T cell activation | 7.53E-13 |
| MF_FAT | GO:0030695~GTPase regulator activity | 6.46E-12 |
| KEGG_PATHWAY | hsa04514:Cell adhesion molecules (CAMs) | 1.78E-11 |
| KEGG_PATHWAY | hsa04640:Hematopoietic cell lineage | 4.85E-09 |
| MF_FAT | GO:0005509~calcium ion binding | 7.21E-08 |
| KEGG_PATHWAY | hsa04060:Cytokine-cytokine receptor interaction | 1.44E-07 |
| KEGG_PATHWAY | hsa04672:Intestinal immune network for IgA production | 1.55E-06 |
| KEGG_PATHWAY | hsa05416:Viral myocarditis | 4.32E-06 |
| KEGG_PATHWAY | hsa05330:Allograft rejection | 9.88E-06 |
| KEGG_PATHWAY | hsa04940:Type I diabetes mellitus | 2.85E-05 |
| CC_FAT | GO:0042611~MHC protein complex | 7.36E-05 |
| KEGG_PATHWAY | hsa05332:Graft-versus-host disease | 3.34E-05 |
| KEGG_PATHWAY | hsa05310:Asthma | 1.90E-04 |
| KEGG_PATHWAY | hsa05340:Primary immunodeficiency | 5.05E-04 |
| KEGG_PATHWAY | hsa05320:Autoimmune thyroid disease | 4.71E-04 |
| **“common-specific aging genes”** | | |
| **Genes decreased expression with age** | | |
| **Category** | **Term** | **Adjusted p-value** |
| BP_FAT | GO:0006350~transcription | 5.26E-23 |
| MF_FAT | GO:0008270~zinc ion binding | 1.68E-21 |
| MF_FAT | GO:0046914~transition metal ion binding | 6.46E-15 |
| MF_FAT | GO:0003677~DNA binding | 7.44E-15 |
| CC_FAT | GO:0031981~nuclear lumen | 8.35E-15 |
| BP_FAT | GO:0051252~regulation of RNA metabolic process | 5.08E-10 |
| BP_FAT | GO:0006355~regulation of transcription, DNA-dependent | 7.08E-10 |
| CC_FAT | GO:0005730~nucleolus | 1.37E-08 |
| MF_FAT | GO:0043167~ion binding | 5.19E-07 |
| MF_FAT | GO:0003723~RNA binding | 4.90E-06 |
| BP_FAT | GO:0008104~protein localization | 5.24E-06 |
| CC_FAT | GO:0016604~nuclear body | 7.01E-06 |
| BP_FAT | GO:0044265~cellular macromolecule catabolic process | 1.52E-05 |
| BP_FAT | GO:0015031~protein transport | 1.76E-05 |
| BP_FAT | GO:0019941~modification-dependent protein catabolic process | 2.85E-05 |
| BP_FAT | GO:0043632~modification-dependent macromolecule catabolic process | 2.85E-05 |
| BP_FAT | GO:0051603~proteolysis involved in cellular protein catabolic process | 2.96E-05 |
| BP_FAT | GO:0044257~cellular protein catabolic process | 3.07E-05 |
| CC_FAT | GO:0016607~nuclear speck | 1.95E-04 |
| **Genes increased expression with age** | | |
| **Category** | **Term** | **Adjusted p-value** |
| CC_FAT | GO:0044459~plasma membrane part | 0.0140 |
| **“healthy-specific aging genes”** | | |
| **Genes decreased expression with age** | | |
| **Category** | **Term** | **Adjusted p-value** |
| CC_FAT | GO:0030529~ribonucleoprotein complex | 2.23E-22 |
| BP_FAT | GO:0006412~translation | 2.61E-20 |
| CC_FAT | GO:0031974~membrane-enclosed lumen | 6.67E-21 |
| KEGG_PATHWAY | hsa03010:Ribosome | 2.91E-17 |
| CC_FAT | GO:0005739~mitochondrion | 1.05E-12 |
| BP_FAT | GO:0006396~RNA processing | 1.35E-06 |
| BP_FAT | GO:0034470~ncRNA processing | 2.61E-05 |
| BP_FAT | GO:0006364~rRNA processing | 5.92E-04 |
| BP_FAT | GO:0016072~rRNA metabolic process | 0.0010 |
| BP_FAT | GO:0006399~tRNA metabolic process | 0.0017 |
| BP_FAT | GO:0006915~apoptosis | 0.0263 |
| **Genes increased expression with age** | | |
| **Category** | **Term** | **Adjusted p-value** |
| MF_FAT | GO:0046914~transition metal ion binding | 6.90E-04 |
| MF_FAT | GO:0008270~zinc ion binding | 0.0026 |
| MF_FAT | GO:0043167~ion binding | 0.0037 |
| MF_FAT | GO:0046872~metal ion binding | 0.0043 |
| MF_FAT | GO:0043169~cation binding | 0.0047 |

**Table S11c. GO functional annotation in aorta artery:** The first column shows the original database/resource where the terms orient. The second column shows the enriched terms associated with genes up-/down-regulated with age. The third column shows the Benjamini-Hochberg modified statistic for a p-value. Only selected top enriched terms with a p-value less than 5% are shown. Gene lists were classified into “core-aging genes”, “common-specific aging genes” and “healthy-specific aging genes”.

| **“core-aging genes”** | | |
| --- | --- | --- |
| **Genes decreased expression with age** | | |
| **Category** | **Term** | **Adjusted p-value** |
| BP_FAT | GO:0006350~transcription | 2.52E-19 |
| MF_FAT | GO:0008270~zinc ion binding | 5.54E-16 |
| MF_FAT | GO:0046914~transition metal ion binding | 1.27E-13 |
| MF_FAT | GO:0003677~DNA binding | 2.95E-13 |
| BP_FAT | GO:0006355~regulation of transcription, DNA-dependent | 4.57E-12 |
| BP_FAT | GO:0051252~regulation of RNA metabolic process | 1.67E-11 |
| KEGG_PATHWAY | hsa00280:Valine, leucine and isoleucine degradation | 3.16E-06 |
| KEGG_PATHWAY | hsa00310:Lysine degradation | 3.83E-04 |
| KEGG_PATHWAY | hsa00640:Propanoate metabolism | 0.0045 |
| BP_FAT | GO:0016568~chromatin modification | 0.0029 |
| MF_FAT | GO:0030528~transcription regulator activity | 0.0034 |
| KEGG_PATHWAY | hsa00071:Fatty acid metabolism | 0.0071 |
| KEGG_PATHWAY | hsa04270:Vascular smooth muscle contraction | 0.0158 |
| KEGG_PATHWAY | hsa04020:Calcium signaling pathway | 0.0176 |
| **Genes increased expression with age** | | |
| **Category** | **Term** | **Adjusted p-value** |
| CC_FAT | GO:0005783~endoplasmic reticulum | 3.21E-23 |
| CC_FAT | GO:0005794~Golgi apparatus | 9.81E-14 |
| BP_FAT | GO:0016192~vesicle-mediated transport | 2.15E-11 |
| BP_FAT | GO:0045184~establishment of protein localization | 1.04E-10 |
| BP_FAT | GO:0015031~protein transport | 1.51E-10 |
| BP_FAT | GO:0010498~proteasomal protein catabolic process | 1.02E-07 |
| BP_FAT | GO:0070727~cellular macromolecule localization | 1.08E-07 |
| CC_FAT | GO:0031982~vesicle | 1.43E-07 |
| KEGG_PATHWAY | hsa03050:Proteasome | 4.67E-06 |
| KEGG_PATHWAY | hsa00010:Glycolysis / Gluconeogenesis | 0.0013 |
| KEGG_PATHWAY | hsa00510:N-Glycan biosynthesis | 0.0034 |
| KEGG_PATHWA | hsa04115:p53 signaling pathway | 0.0066 |
| KEGG_PATHWAY | hsa05110:Vibrio cholerae infection | 0.0068 |
| KEGG_PATHWAY | hsa04142:Lysosome | 0.0069 |
| **“common-specific aging genes”** | | |
| **Genes decreased expression with age** | | |
| **Category** | **Term** | **Adjusted p-value** |
| MF_FAT | GO:0043167~ion binding | 0.0127 |
| MF_FAT | GO:0043169~cation binding | 0.0159 |
| MF_FAT | GO:0008270~zinc ion binding | 0.0167 |
| MF_FAT | GO:0046872~metal ion binding | 0.0205 |
| MF_FAT | GO:0046914~transition metal ion binding | 0.0276 |
| **Genes increased expression with age** | | |
| **Category** | **Term** | **Adjusted p-value** |
| MF_FAT | GO:0003779~actin binding | 2.26E-05 |
| MF_FAT | GO:0008092~cytoskeletal protein binding | 7.79E-05 |
| KEGG_PATHWAY | hsa05016:Huntington's disease | 0.0080 |
| CC_FAT | GO:0030016~myofibril | 0.0165 |
| KEGG_PATHWAY | hsa04144:Endocytosis | 0.0167 |
| CC_FAT | GO:0005747~mitochondrial respiratory chain complex I | 0.0169 |
| CC_FAT | GO:0070161~anchoring junction | 0.0185 |
| BP_FAT | GO:0048193~Golgi vesicle transport | 0.0210 |
| KEGG_PATHWAY | hsa00190:Oxidative phosphorylation | 0.0212 |
| CC_FAT | GO:0070469~respiratory chain | 0.0227 |
| BP_FAT | GO:0015031~protein transport | 0.0281 |
| **“healthy-specific aging genes”** | | |
| **Genes decreased expression with age** | | |
| **Category** | **Term** | **Adjusted p-value** |
| CC_FAT | GO:0043292~contractile fiber | 7.11E-04 |
| CC_FAT | GO:0030016~myofibril | 0.0011 |
| CC_FAT | GO:0030017~sarcomere | 0.0012 |
| CC_FAT | GO:0030018~Z disc | 0.0020 |
| CC_FAT | GO:0031674~I band | 0.0040 |

**Table S11d. GO functional annotation in lung:** The first column shows the original database/resource where the terms orient. The second column shows the enriched terms associated with genes up-/down-regulated with age. The third column shows the Benjamini-Hochberg modified statistic for a p-value. Only selected top enriched terms with a p-value less than 5% are shown. Gene lists were classified into “core-aging genes”, “common-specific aging genes” and “healthy-specific aging genes”.

| **“common-specific aging genes”** | | |
| --- | --- | --- |
| **Genes decreased expression with age** | | |
| **Category** | **Term** | **Adjusted p-value** |
| CC_FAT | GO:0005694~chromosome | 1.29E-11 |
| BP_FAT | GO:0022403~cell cycle phase | 1.36E-11 |
| BP_FAT | GO:0000279~M phase | 1.10E-10 |
| BP_FAT | GO:0000278~mitotic cell cycle | 3.09E-10 |
| BP_FAT | GO:0000280~nuclear division | 4.09E-10 |
| BP_FAT | GO:0006974~response to DNA damage stimulus | 1.11E-07 |
| BP_FAT | GO:0006259~DNA metabolic process | 3.08E-07 |
| BP_FAT | GO:0006260~DNA replication | 1.15E-06 |
| CC_FAT | GO:0005657~replication fork | 3.21E-06 |
| BP_FAT | GO:0006281~DNA repair | 5.01E-06 |
| BP_FAT | GO:0051301~cell division | 3.31E-05 |
| BP_FAT | GO:0000075~cell cycle checkpoint | 4.05E-04 |
| KEGG_PATHWAY | hsa04110:Cell cycle | 6.27E-04 |
| KEGG_PATHWAY | hsa03030:DNA replication | 9.35E-04 |
| KEGG_PATHWAY | hsa03430:Mismatch repair | 0.0038 |
| **Genes increased expression with age** | | |
| **Category** | **Term** | **Adjusted p-value** |
| CC_FAT | GO:0005576~extracellular region | 3.19E-04 |
| CC_FAT | GO:0031012~extracellular matrix | 9.26E-04 |
| CC_FAT | GO:0005578~proteinaceous extracellular matrix | 8.43E-04 |
| BP_FAT | GO:0001501~skeletal system development | 0.0014 |
| MF_FAT | GO:0043167~ion binding | 0.0044 |
| MF_FAT | GO:0043169~cation binding | 0.0096 |
| MF_FAT | GO:0046872~metal ion binding | 0.0097 |

**Table S12. Comparison of aging genes and GenAge.**

Here we performed a comparison of the estimated age-related genes in the current study to GenAge in subcutaneous fat, aorta artery, tibial artery and lung. No. of background genes represents the number of selected genes we used to identify age-related genes in each tissue; No. of GenAge genes is the number of human aging genes from GenAge; No. of CAGs/CSAGs/HSAGs genes is the number of age-associated genes identified from “common” cohort and “healthy” cohort; No. of overlapped genes is the number of overlapped genes between three gene sets and GenAge respectively; p-value is calculated by using hypergeometric test.

| Tissues | No. of background genes | No. of GenAge | CAGs | | | CSAGs | | | HSAGs | | |
| --- | --- | --- | --- | --- | --- | --- | --- | --- | --- | --- | --- |
|  |  |  | No. of CAGs | No. of overlapped genes | p-value | No. of CSAGs | No. of overlapped genes | p-value | No. of HSAGs | No. of overlapped genes | p-value |
| Subcutaneous Fat | 18,643 | 282 | 2,177 | 54 | 1.63e-04 | 1635 | 24 | 0.59 | 189 | 3 | 0.55 |
| Aorta Artery | 18,189 | 281 | 4,319 | 91 | 5.65e-04 | 1505 | 24 | 0.47 | 389 | 10 | 0.08 |
| Tibial Artery | 17,708 | 278 | 5,487 | 92 | 0.24 | 3221 | 53 | 0.38 | 2686 | 28 | 1.00 |
| Lung | 19,540 | 285 | 96 | 1 | 0.76 | 884 | 22 | 0.01 | 11 | 0 | 1.00 |
